# Non-estrogenic Xanthohumol Derivatives Mitigate Insulin Resistance and Cognitive Impairment in High-Fat Diet-induced Obese Mice

**DOI:** 10.1038/s41598-017-18992-6

**Published:** 2018-01-12

**Authors:** Cristobal L. Miranda, Lance A. Johnson, Oriane de Montgolfier, Valerie D. Elias, Lea S. Ullrich, Joshua J. Hay, Ines L. Paraiso, Jaewoo Choi, Ralph L. Reed, Johana S. Revel, Chrissa Kioussi, Gerd Bobe, Urszula T. Iwaniec, Russell T. Turner, Benita S. Katzenellenbogen, John A. Katzenellenbogen, Paul R. Blakemore, Adrian F. Gombart, Claudia S. Maier, Jacob Raber, Jan F. Stevens

**Affiliations:** 1 Linus Pauling Institute, Oregon State University, Corvallis, OR 97331 USA; 2 Department of Pharmaceutical Sciences, Oregon State University, Corvallis, OR 97331 USA; 3 Department of Behavioral Neuroscience, Oregon Health & Science University, Portland, OR 97239 USA; 4 Department of Animal & Rangeland Sciences, Oregon State University, Corvallis, OR 97331 USA; 5 Skeletal Biology Laboratory, School of Biological and Population Health Sciences, College of Public Health and Human Sciences, Oregon State University, Corvallis, OR 97331 USA; 6 Departments of Molecular & Integrative Physiology, University of Illinois at Urbana-Champaign, Urbana, IL 61801 USA; 7 Department of Chemistry, University of Illinois at Urbana-Champaign, Urbana, IL 61801 USA; 8 Department of Chemistry, Oregon State University, Corvallis, OR 97331 USA; 9 Department of Biochemistry & Biophysics, Oregon State University, Corvallis, OR 97331 USA; 10 Departments of Neurology and Radiation Medicine, Division of Neuroscience, Oregon National Primate Research Center, Oregon Health & Science University, Beaverton, OR 97006 USA

## Abstract

Xanthohumol (XN), a prenylated flavonoid from hops, improves dysfunctional glucose and lipid metabolism in animal models of metabolic syndrome (MetS). However, its metabolic transformation into the estrogenic metabolite, 8-prenylnaringenin (8-PN), poses a potential health concern for its use in humans. To address this concern, we evaluated two hydrogenated derivatives, α,β-dihydro-XN (DXN) and tetrahydro-XN (TXN), which showed negligible affinity for estrogen receptors α and β, and which cannot be metabolically converted into 8-PN. We compared their effects to those of XN by feeding C57BL/6J mice a high-fat diet (HFD) containing XN, DXN, or TXN for 13 weeks. DXN and TXN were present at higher concentrations than XN in plasma, liver and muscle. Mice administered XN, DXN or TXN showed improvements of impaired glucose tolerance compared to the controls. DXN and TXN treatment resulted in a decrease of HOMA-IR and plasma leptin. C2C12 embryonic muscle cells treated with DXN or TXN exhibited higher rates of uncoupled mitochondrial respiration compared to XN and the control. Finally, XN, DXN, or TXN treatment ameliorated HFD-induced deficits in spatial learning and memory. Taken together, DXN and TXN could ameliorate the neurocognitive-metabolic impairments associated with HFD-induced obesity without risk of liver injury and adverse estrogenic effects.

## Introduction

Metabolic syndrome (MetS), a major risk factor for cardiovascular disease and type 2 diabetes (T2D), is clinically diagnosed by two or more of the following conditions: abdominal obesity, hypertriglyceridemia, low high-density lipoprotein (HDL) level, hypertension, and hyperglycemia^1^. Accumulating evidence suggests that MetS and its components are also associated with cognitive dysfunction and dementia^2–6^. Diets high in saturated fat induce chronic low-grade inflammation that contributes to the development of MetS^7^. Obesity and consumption of a high-fat diet (HFD) can impair cognitive function^8^, including hippocampus-dependent memory in humans^9,10^. In humans, risk factors for cognitive decline and dementia include obesity^11^, insulin resistance^12–14^, type-2 diabetes (T2D)^15,16^ and consumption of a HFD^8,17–21^. MetS has reached epidemic proportions, with an estimated prevalence of 35% among U.S. adults in 2012^22^, but no single agent is effective in treating it, and many of the various drugs prescribed have significant risks of adverse effects. For example, biguanides such as metformin are widely-prescribed drugs for treating T2D and MetS. Metformin appears to improve insulin signaling by activating 5′-AMP-activated protein kinase (AMPK), but there is no convincing evidence of its effects on dysfunctional lipid metabolism at therapeutic doses^23^. Furthermore, the Diabetes Prevention Program recently published an association between metformin use for pre-T2D and low vitamin B12 levels^24^. Another concern with metformin is lactic acidosis when renal function is poor^25^, and therefore metformin should not be used in this population. The recently developed pan-AMPK activator MK-8722 improves glucose homeostasis but induces cardiac hypertrophy and increases cardiac glycogen in rodents and rhesus monkeys^26^. Thiazolidinediones (glitazones) are peroxisome proliferator-activated receptor-γ (PPARγ) agonists, that promote *de novo* lipogenesis (DNL) and can exert long-term adverse side effects including increased body weight and cardiovascular toxicity^27^. A meta-analysis of 42 trials linked the PPARγ agonist rosiglitazone (Avandia^®^) to a 43% increased risk of heart attack (*p* = 0.03)^28,29^. These findings strongly underscore the need for safer alternative intervention agents.

**Figure 1:**
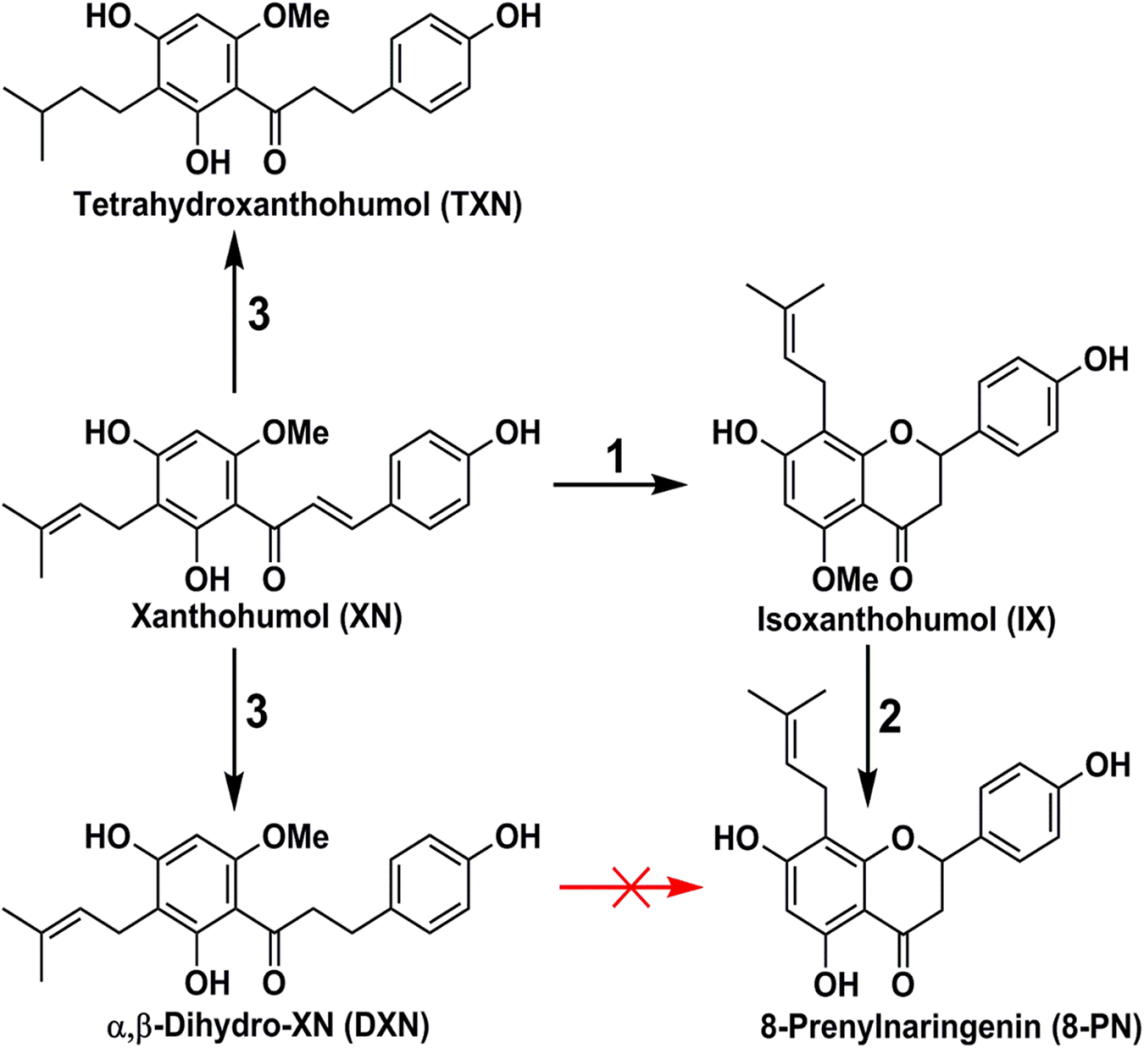
Structures of xanthohumol (XN) and its metabolites and derivatives. Steps: 1, spontaneous cyclization by intramolecular Michael-type addition; 2, hepatic or gut microbial *O*-demethylation; 3, chemical synthesis.

Rodents are widely used in preclinical studies of MetS and obesity^30^. Mice fed a HFD show hippocampus-dependent memory deficits, while rats fed a HFD demonstrated impaired hippocampal function and disruptions in the blood–brain barrier^10,11,31^. Xanthohumol (XN; 3′-[3,3-dimethyl allyl]-2′,4′,4-trihydroxy-6′-methoxychalcone, Fig. 1), the principal prenylated flavonoid found in hops (*Humulus lupulus*), exerts anti-obesity effects in Zucker rats^32,33^ and in various mouse strains^34–36^. In our previous work^36^, we demonstrated that XN has bioactivities potentially useful for countering the metabolic aberrations of MetS. Our studies have shown that treating HFD-fed C57BL/6 J mice orally with XN (60 mg/kg/day) reduced their plasma low-density lipoprotein-cholesterol (LDL-c, −80%), interleukin-6 (IL-6, −78%), HOMA-IR (−52%), leptin (−41%), and plasma levels of the LDL receptor-degrading enzyme proprotein convertase subtilisin/kexin type 9 (PCSK9) (−44%) levels compared to those of vehicle/HFD controls^36^. XN treatment can lower LDL-c levels by upregulating CYP7A1^35^ (the rate-limiting enzyme that mediates the conversion of cholesterol into bile acids (BAs)) and by farnesoid X receptor (FXR)-mediated downregulation of PCSK9^37^. XN binds to the ligand-binding domain of FXR^38^ and appears to activate farnesoid X receptors (FXR) *in vivo*^35^. FXR is thought to be an attractive therapeutic target for MetS because when activated it inhibits gluconeogenesis and DNL^39^. Unlike the various synthetic and naturally occurring steroidal FXR agonists that have been tested and found to have unacceptable adverse side effects (e.g. guggulsterone, which elevated LDL-c in clinical trials^40^), XN appears to have an excellent safety profile with no detectable toxicity in mice at doses up to at least 1,000 mg/kg^41^. By comparing XN pharmacokinetics and doses between rats^42^ and humans^43^, we established that effective plasma levels of XN are achievable in humans at a single oral daily dose of 180 mg.

The use of XN in dietary supplements raises some concerns, as one of its metabolites, 8-prenylnaringenin (8-PN), is the most potent phytoestrogen known to date^44–47^. Due to the presence of an α,β-unsaturated ketone in its chemical structure, XN can spontaneously form a stable isomer, isoxanthohumol (IX), which intestinal microbiota^48^ and hepatic cytochrome P450 enzymes^49^ can transform into 8-PN. Hydrogenation of XN’s double bond of its α,β-unsaturated ketone moiety prevents the metabolic formation of 8-PN (Fig. 1). Therefore, the development of XN analogues that cannot be converted into 8-PN, thereby eliminating potential side effects with retention of the beneficial effects, would provide attractive treatment options to mitigate MetS and cognitive impairments associated with obesity.

In the current study, we describe the biological activities of two hydrogenated XN derivatives, α,β-dihydro-XN (DXN) and tetrahydro-XN (TXN) (Fig. 1), which lack the α,β-unsaturated ketone and cannot form the estrogenic metabolite 8-PN. To determine the potential of these compounds in preventing impairments induced by a HFD, we administered XN, DXN and TXN orally to C57BL/6J mice for 13 weeks. Based on the findings from our previous work^36^, we selected a dose of 30 mg/kg/day because this dose, which resulted in sub-maximal improvement of MetS-related parameters in XN-treated mice, allows comparison of differences in pharmacological response among XN, DXN, and TXN treatments. We examined tissue and plasma bioavailability, estrogen receptor binding affinity, and the effects of the compounds on mitochondrial function and cognition. Peripheral metabolic markers and glucose tolerance were evaluated, and hippocampus-dependent cognitive performance was assessed using the water maze. In addition, *in vivo* markers of hepatotoxicity, i.e., plasma aspartate aminotransferase (AST) and alanine aminotransferase (ALT), were measured in control and treated mice.

## Results

### XN, DXN and TXN show very low affinity for the estrogen receptors (ER)α and ERβ and do not induce cell proliferation or estrogen-dependent progesterone receptor (PGR) gene expression in MCF-7 cells

**Figure 2:**
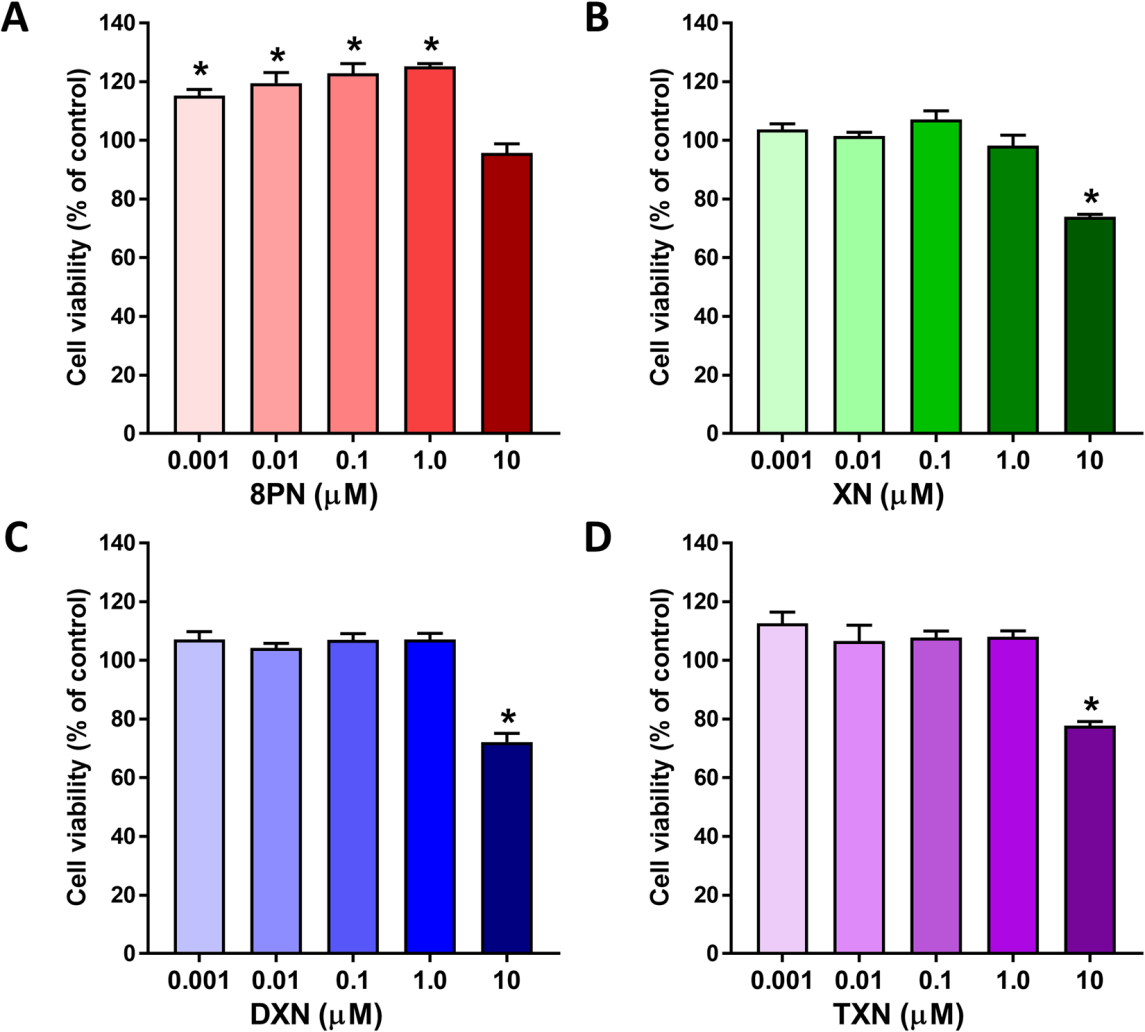
Cell viability/proliferation of MCF-7 cells after a 48-h exposure to 8-PN (**A**), XN (**B**), DXN (**C**), or TXN (**D**). Low concentrations (0.001 to 1 µM) of XN, DXN and TXN did not significantly increase cell viability/proliferation of MCF-7 cells whereas high concentrations (10 µM) of these compounds significantly decreased cell viability/proliferation. Data are represented as mean ± SEM of 6 replicate wells. Asterisk denotes significantly different from control, *p* < 0.05 by ANOVA.

**Figure 3:**
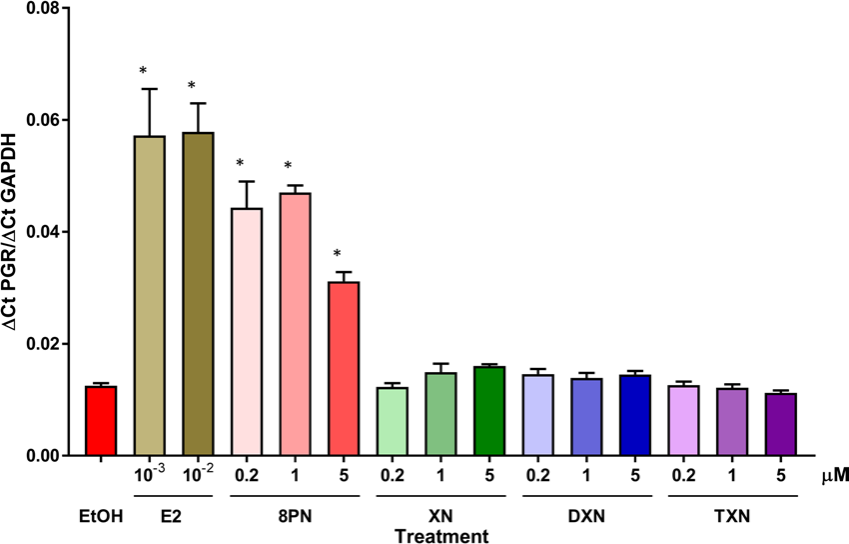
XN, DXN and TXN do not induce progesterone receptor (PGR) expression in MCF-7 cells. Cells were treated with 0.1% ethanol (EtOH), estradiol (E2, 1 and 10 nM), 8-PN (0.2, 1 and 5 μM), or XN and its derivatives, DXN and TXN, for 20 h. PGR expression was determined by quantitative real-time PCR. A statistically significant induction of PGR expression was observed with both concentrations of estradiol and all three concentrations of 8-PN, but not by XN, DXN or TXN. Data are represented as mean ± SD of 3 replicate experiments. Asterisk denotes significantly different from control (EtOH), *p* < 0.05 by ANOVA.

We measured the affinity of XN and its derivatives for estrogen receptors using radiolabeled ligand binding assays. XN, DXN and TXN had very low binding to both ERα and ERβ (Table 1). In contrast, 8-PN exhibited >100-fold greater affinity for both receptors compared to XN, DXN, and TXN. These data indicate that, compared to estradiol, the hydrogenated XN derivatives possess negligible intrinsic estrogenic activity. We measured MCF-7 cell viability after 48 h exposure to different concentrations of 8-PN, XN, DXN or TXN (Fig. 2). The potent phytoestrogen, 8-PN, induced cell proliferation at concentrations up to 1 µM (Fig. 2A). By contrast, XN (Fig. 2B), DXN (Fig. 2C), and TXN (Fig. 2D) in a concentration range of 0.001–1 µM for 48 h did not produce a significant increase in cell viability/proliferation, suggesting the lack of estrogenicity of these compounds. At 10 µM, all four compounds reduced the cell viability of MCF-7 cells (Fig. 2A–D). Consistent with these results, we did not observe induction of expression of PGR, an ER-target gene, by XN, DXN and TXN, but PGR expression was induced by estradiol and 8-PN (Fig. 3). Taken together these data indicate that XN, DXN and TXN do not possess estrogenic activity like 8-PN.

**Table 1:** ERα and ERβ binding affinities (relative binding affinity [RBA]) for XN, DXN, TXN, and 8-PN.

Compound	RBA ERα	RBA ERβ
Xanthohumol (XN)	0.0021 ± 0.0003	0.003 ± 0.001
Isoxanthohumol (IX)	0.0020 ± 0.0002	0.019 ± 0.004
α,β-Dihydro-XN (DXN)	0.0029 ± 0.0007	0.009 ± 0.002
Tetrahydro-XN (TXN)	0.0033 ± 0.001	0.009 ± 0.002
8-Prenylnaringenin (8-PN)	0.686 ± 0.18	2.42 ± 0.62

Relative binding affinity (RBA) values are determined by competitive radiometric binding assays and are expressed as IC_50_^[estradiol]^/IC50^[compound]^ × 100 (RBA, estradiol = 100). In these assays, the *K*d for estradiol is 0.2 nM on ERα and 0.5 nM on ERβ.

### Effects of XN, DXN and TXN on body weight gain, food intake and metabolic parameters

**Figure 4:**
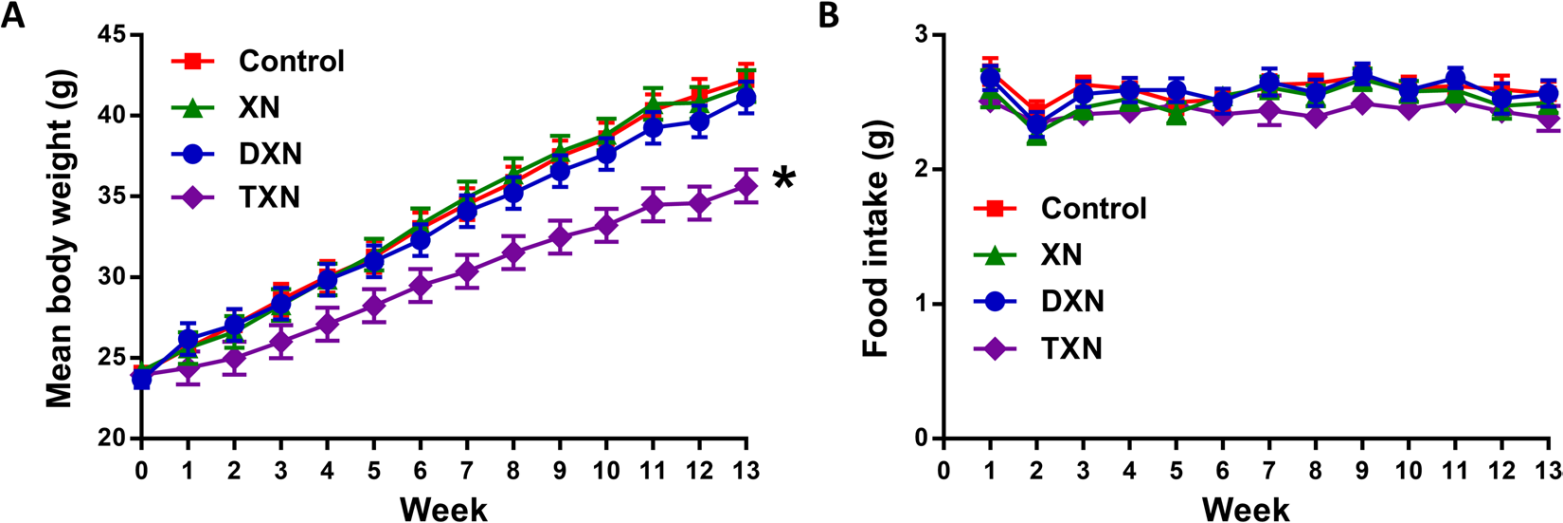
Weekly body weights (**A**) and weekly food intake (**B**) of male mice fed a HFD with no XN/DXN/TXN (control), with 30 mg XN/kg body weight/day (XN), 30 mg DXN/kg body weight/day (DXN), or 30 mg TXN/kg body weight/day (TXN) for 13 weeks. Values are expressed as mean ± SEM of 12 mice per group (11 for the TXN group). Asterisk denotes significantly different from control group, *p* < 0.05 by ANOVA.

Compared to the HFD-fed, vehicle-treated control group, mice supplemented with TXN gained significantly less body weight (Fig. 4A, Table 2). There was no change in body weight gain of mice in the XN or DXN treatment groups compared to the vehicle-treated control group. Food intake remained unchanged from controls during the course of XN, DXN or TXN treatment (Fig. 4B, Table 2). Only TXN treatment caused a reduction in feed efficiency (calculated as g body weight gain divided by total food intake, Table 2) that would explain the reduced body weight gain in this group. Liver weight (−21%), plasma glucose (−25%), insulin (−82%), and leptin concentrations (−44%) were significantly lower in the TXN treatment group compared with controls (Table 1). DXN also significantly decreased plasma leptin concentrations (−26%; Table 2). Both DXN and TXN produced a significant reduction in HOMA-IR index of mice fed a HFD, with a more profound reduction in the TXN-treated group (13% of control mice, Table 2). Treatment of mice with XN, DXN or TXN did not cause an increase in plasma AST and ALT as compared to control mice (Table 2), suggesting that the test compounds were not hepatotoxic *in vivo*. Plasma ALT activities were significantly reduced by treatment with XN, DXN or TXN which may indicate that these compounds are hepatoprotective in mice fed a HFD. Feeding a HFD to C57BL/6J mice has been shown to increase serum ALT and AST levels^50,51^, with ALT being well-known to be more specific for liver damage than AST.

**Table 2:** Body weight gain, food intake, liver weight and metabolic parameters of mice fed experimental diets.

Parameter	Control	XN	DXN	TXN
Body weight gain (g)	16.6 ± 1.3	16.1 ± 1.3	15.1 ± 1.3	9.70 ± 1.4*
Food intake (g/day)	2.69 ± 0.09	2.59 ± 0.09	2.66 ± 0.09	2.51 ± 0.09
Feed efficiency (g weight gain/g food intake)	0.068 ± 0.006	0.068 ± 0.006	0.062 ± 0.006	0.042 ± 0.006*
Liver weight (g)	1.17 ± 0.07	1.14 ± 0.07	1.11 ± 0.07	0.93 ± 0.07*
Fasting plasma glucose (mg/dL)	163 ± 7.4	169 ± 8.3	150 ± 13.0	118 ± 7.2*
Plasma triglycerides (mg/dL)	87.5 ± 6.6	80.6 ± 4.2	84.4 ± 4.9	79.3 ± 7.8
Plasma cholesterol (mg/dL)	177 ± 8.5	196 ± 8.5	167 ± 8.5	167 ± 8.9
Plasma insulin (ng/mL)	2.70 ± 0.76	2.61 ± 0.65	2.08 ± 0.69	0.49 ± 0.08*
HOMA-IR	32.2 ± 1.5	31.3 ± 1.7	22.0 ± 5.2*	4.10 ± 0.3*
Plasma leptin (ng/mL)	11.4 ± 1.00	10.6 ± 1.00	8.48 ± 1.00*	6.39 ± 1.04*
Plasma AST (U/L)	109.9 ± 8.6	112.3 ± 3.3	105.6 ± 6.5	110.5 ± 6.0
Plasma ALT (U/L)	92.4 ± 9.5	68.8 ± 6.8*	55.8 ± 6.1*	56.8 ± 10.5*

All values are means ± SE of 11–12 animals. *p < 0.05 versus control.

### Steady-state plasma, liver and muscle concentrations of DXN and TXN are higher compared to XN levels

**Figure 5:**
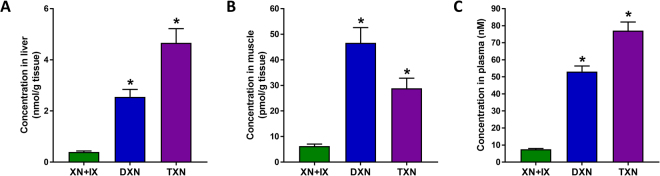
Liver, muscle and plasma concentrations of XN, DXN, and TXN. Concentrations of these compounds were examined by UPLC-MS/MS in the (**A**) liver, (**B**) muscle and (**C**) plasma of mice fed a HFD for 13 weeks. Values are mean ± SEM. *p < 0.05 compared to XN + IX, ANOVA, n = 11–12/treatment.

We measured the concentrations of XN + IX, DXN and TXN in plasma, liver, and hind leg skeletal muscle of mice after 13 weeks of feeding with a HFD containing XN, DXN, or TXN (to deliver a daily dose of 30 mg/kg body weight). The concentrations of DXN and TXN were 6- and 12-fold greater in liver, respectively (Fig. 5A; both *p* < 0.0001), were 7-fold and 5-fold greater in muscle, respectively (Fig. 5B, both *p* < 0.0001), and were 7-fold and 10-fold greater in plasma, respectively (Fig. 5C; both *p* < 0.0001), compared with the combined levels of XN and its spontaneously formed isomerization product, IX (indicated as XN + IX in Fig. 5).

### XN, DXN and TXN improve peripheral glucose metabolism in HFD-fed mice

**Figure 6:**
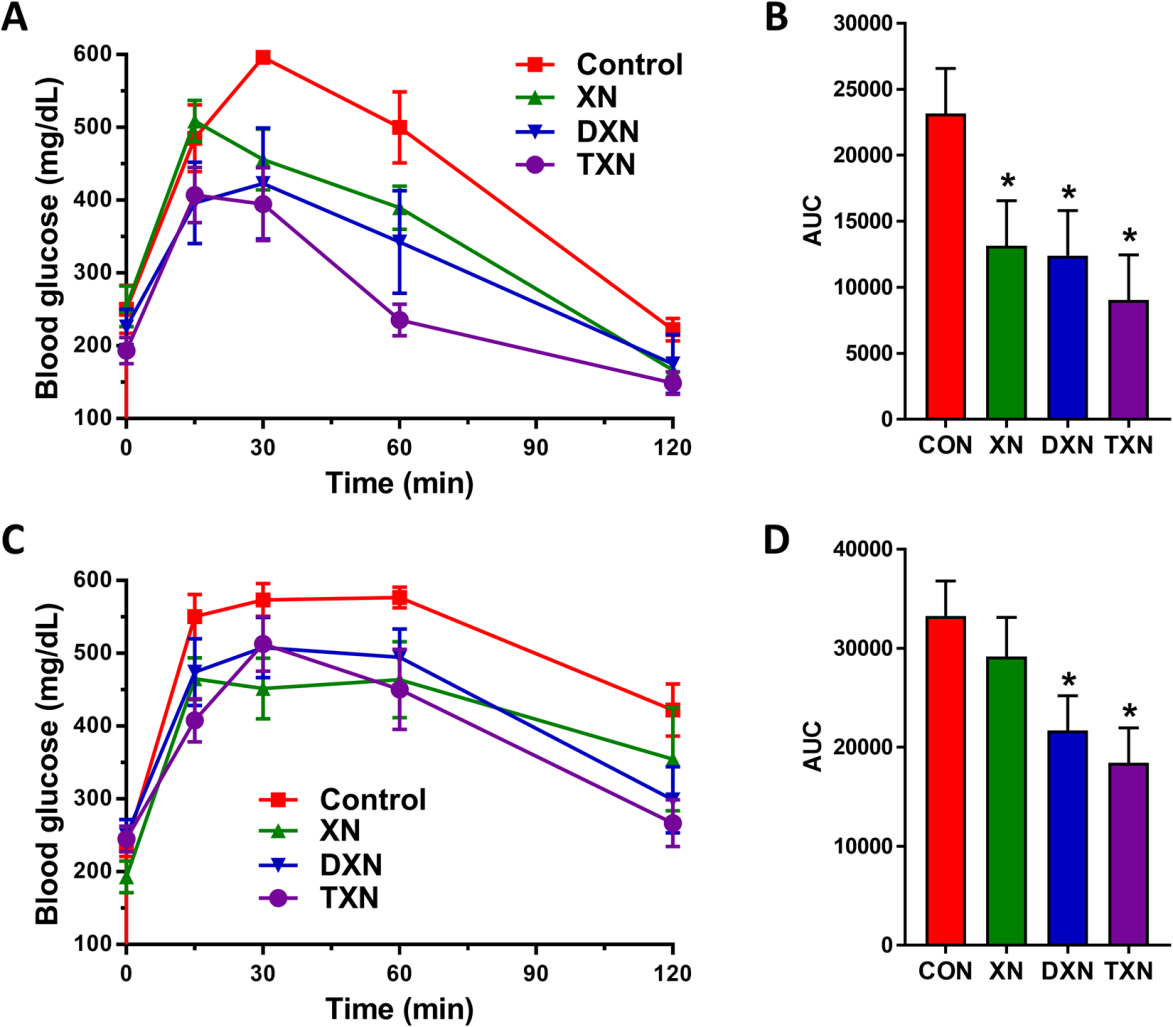
Effect of XN, DXN, and TXN supplementation on glucose tolerance in mice fed a HFD. Blood glucose levels were measured in mice after 4 (**A**,**B**) and 11 (**C**,**D**) weeks of feeding. The mice were fasted for 6 h before i.p. injection of glucose, 2 g/kg body weight, and then blood was analyzed for glucose at designated times. Bar graphs on the right represent areas under the curve (AUC) shown on the left. *p < 0.05 compared to control (ANOVA), n = 5/treatment.

We examined the effects of XN and its derivatives on peripheral glucose metabolism in mice fed a HFD. All three compounds improved glucose clearance in HFD-fed mice at both 4 weeks (Fig. 6A,B; the area under the curve (AUC) decreased by 43%, 46%, and 61% following XN, DXN, and TXN treatment, respectively, compared with control) and, except for XN, 11 weeks (Fig. 6C,D; AUC decreased by 12% (not significantly different from controls), 35%, and 45% following XN, DXN, and TXN treatment, respectively, compared with vehicle-treated controls).

### XN activates liver AMPK whereas XN, DXN, and TXN inhibit AMPK activation in skeletal muscle

**Figure 7:**
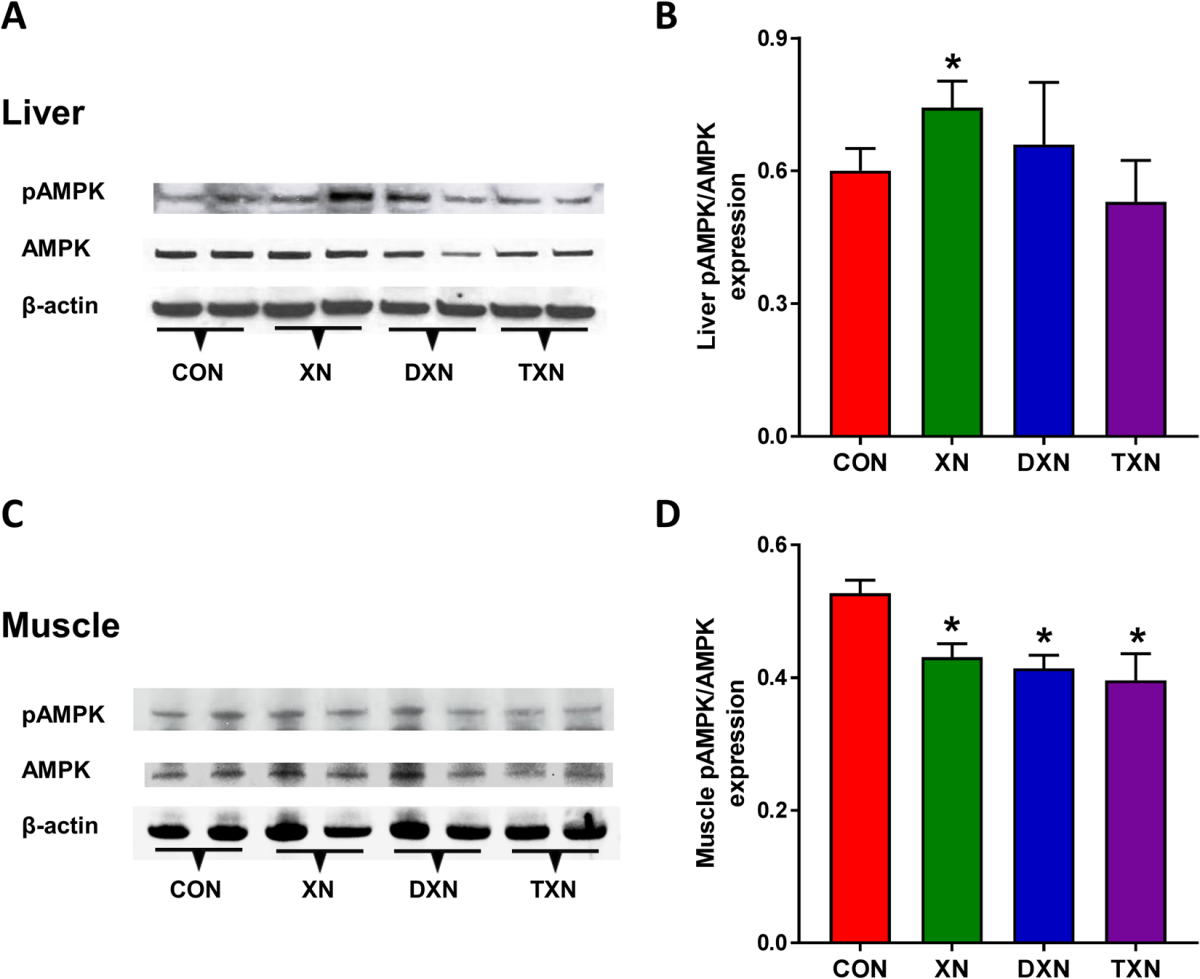
Western blotting analyses of pAMPK, AMPK and β-actin (loading control) in liver (**A**,**B**) and skeletal muscle (**C**,**D**) of mice fed a HFD with or without treatment with XN, DXN and TXN. Protein bands were detected using a chemiluminescence detector (BIO-RAD ChemiDoc MP Imaging System). Band intensities were quantitated using Image J software. Data in bar graphs represent the ratios of pAMPK/AMPK from four samples per group ± SEM. *p < 0.05 compared to control (ANOVA). The represented bands were extracted from independent blots shown in Supplementary Figure 8.

We next examined the effects of XN, DXN and TXN on the activation of AMPK in liver (Fig. 7A,B) and skeletal muscle (Fig. 7C,D) of mice fed HFD. XN treatment increased the activation of liver AMPK by 24% as shown by the ratio of phosphorylated AMPKα/total AMPKα. However, DXN and TXN treatment did not cause a significant activation of liver AMPK. In skeletal muscle, XN, DXN, and TXN decreased the activation of AMPK by 19%, 22%, and 25%, respectively.

### DXN and TXN increase mitochondrial uncoupling in C2C12 cells

**Figure 8:**
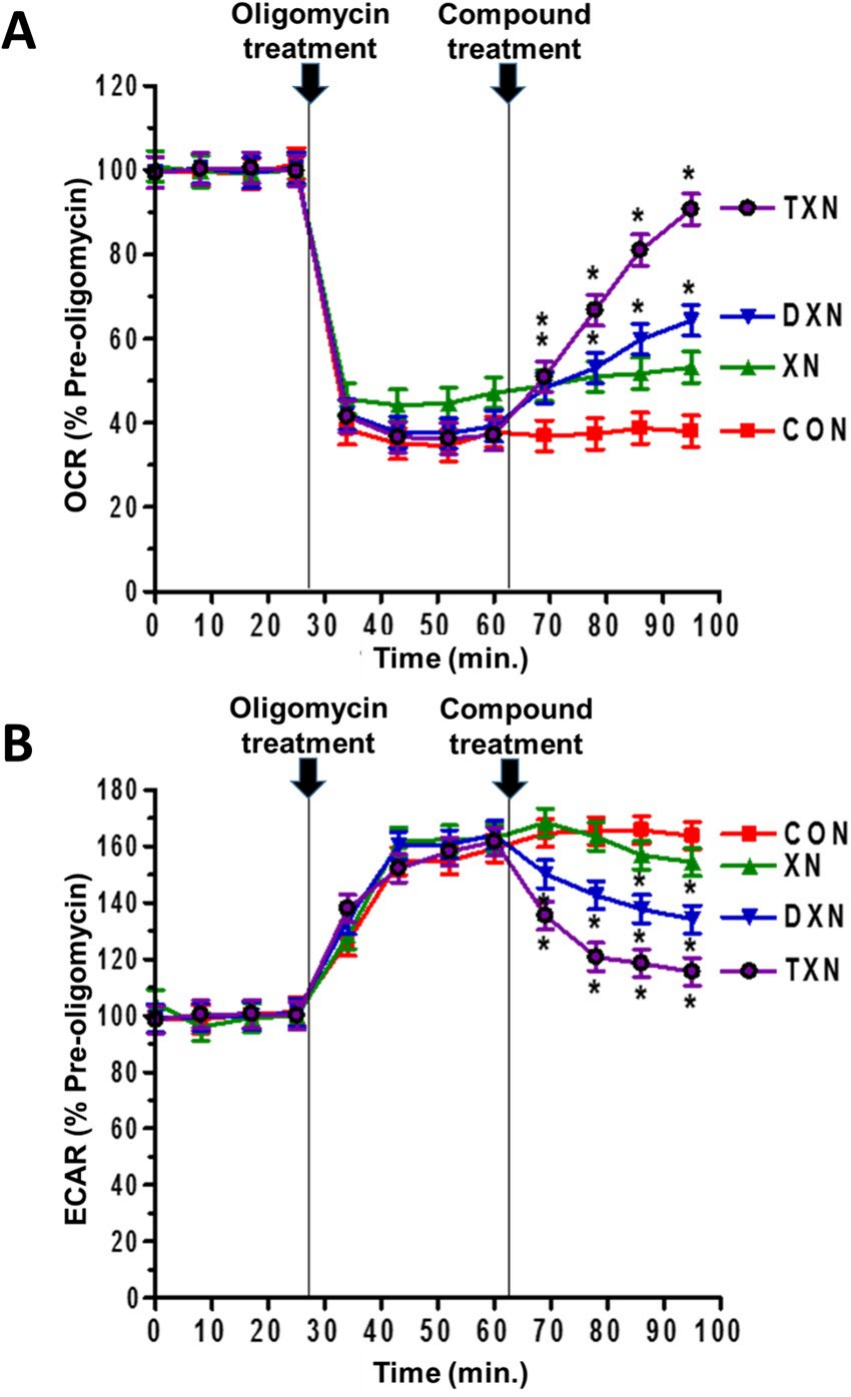
Comparison of XN, DXN and TXN as mild mitochondrial uncouplers. DXN and TXN cause mitochondrial uncoupling in cells. C2C12 cells were sequentially treated with oligomycin (1 μM) and 5 μM of the test compounds while (**A**) the oxygen rate (OCR) and (**B**) the extracellular acidification rate (ECAR) was monitored. *p < 0.05 compared to control by ANOVA (*n* = 5).

To ensure that mitochondrial uncoupling experiments were not confounded by compound-induced alterations in cell viability, we first assessed the viability of cells exposed to the test compounds by using the MTT assay. After 1 h of exposure, XN, DXN and TXN did not significantly affect cell viability up to 50 μM (Supplementary Fig. S1a). Using the same concentrations and exposure periods, we next examined the ability of XN, DXN and TXN to act as mitochondrial uncouplers. Mitochondrial uncoupling is one possible mechanism to increase energy expenditure and prevent obesity^52,53^. To investigate whether the addition of XN, DXN or TXN could stimulate uncoupled mitochondrial respiration in C2C12 cells, we measured the cells’ oxygen consumption rate (OCR) after injection of the ATP synthase inhibitor, oligomycin, into the medium surrounding the cells. Increases in OCR during this period of ATP synthesis inhibition represents uncoupler-linked OCR, and thus reflects the mitochondrial uncoupling effect of each compound. We used a diluted solution of ethanol in the running medium (0.1% final concentration in wells) as a vehicle control. At 5 µM concentration, we observed that DXN and TXN significantly increased the OCR in the presence of oligomycin, by 28.7 ± 6.9% for DXN and by 56.8 ± 6.9% for TXN, compared to the baseline in five independent experiments (Fig. 8A). A concentration of 5 µM appeared to be sufficient for both DXN and TXN to have a significant effect on the OCR while XN itself did not significantly increase the OCR at the same concentration. TXN was the most potent mitochondrial uncoupler among the three compounds.

Treatment of cells with oligomycin inhibits oxidative phosphorylation. To satisfy their energy needs, the cells resort to aerobic glycolysis resulting in cellular excretion of lactic acid into the medium, which we measured as the extracellular acidification rate (ECAR). Following treatment of C2C12 cells with oligomycin, we observed a 1.6-fold increase in ECAR (Fig. 8B). Subsequent addition of XN, DXN or TXN (5 µM final concentration in the medium) resulted in a significant decrease in ECAR for all three compounds (Fig. 8B), consistent with compound-induced utilization of reducing equivalents (NADH) for uncoupled respiration at the expense of NADH-mediated conversion of pyruvate into lactate.

### XN, DXN and TXN do not display cytotoxicity in the human hepatocyte cell line, HepG2, at concentrations attainable in hepatic tissue following oral dosing at 30 mg/kg/day

HepG2 cells were used in the present study to determine the cytotoxicity of XN, DXN, and TXN. This human cell line displays many of the genotypic features of normal liver cells and has been accepted as an *in vitro* model to detect the cytotoxic effects of new drugs and chemicals^54^. Exposure of the cells to XN, DXN, and TXN at concentrations in the range 1–25 µM did not alter cell viability compared to the vehicle-treated control cells (Supplementary Fig. S1b). These findings suggest that oral dosing at 30 mg/kg/day does not cause liver toxicity, as the highest liver concentration measured (TXN, Fig. 5) was 5 µM, thus well within the non-cytotoxic 1–25 µM range.

### Improved spatial learning and memory of HFD mice following treatment with XN or its hydrogenated derivatives

**Figure 9:**
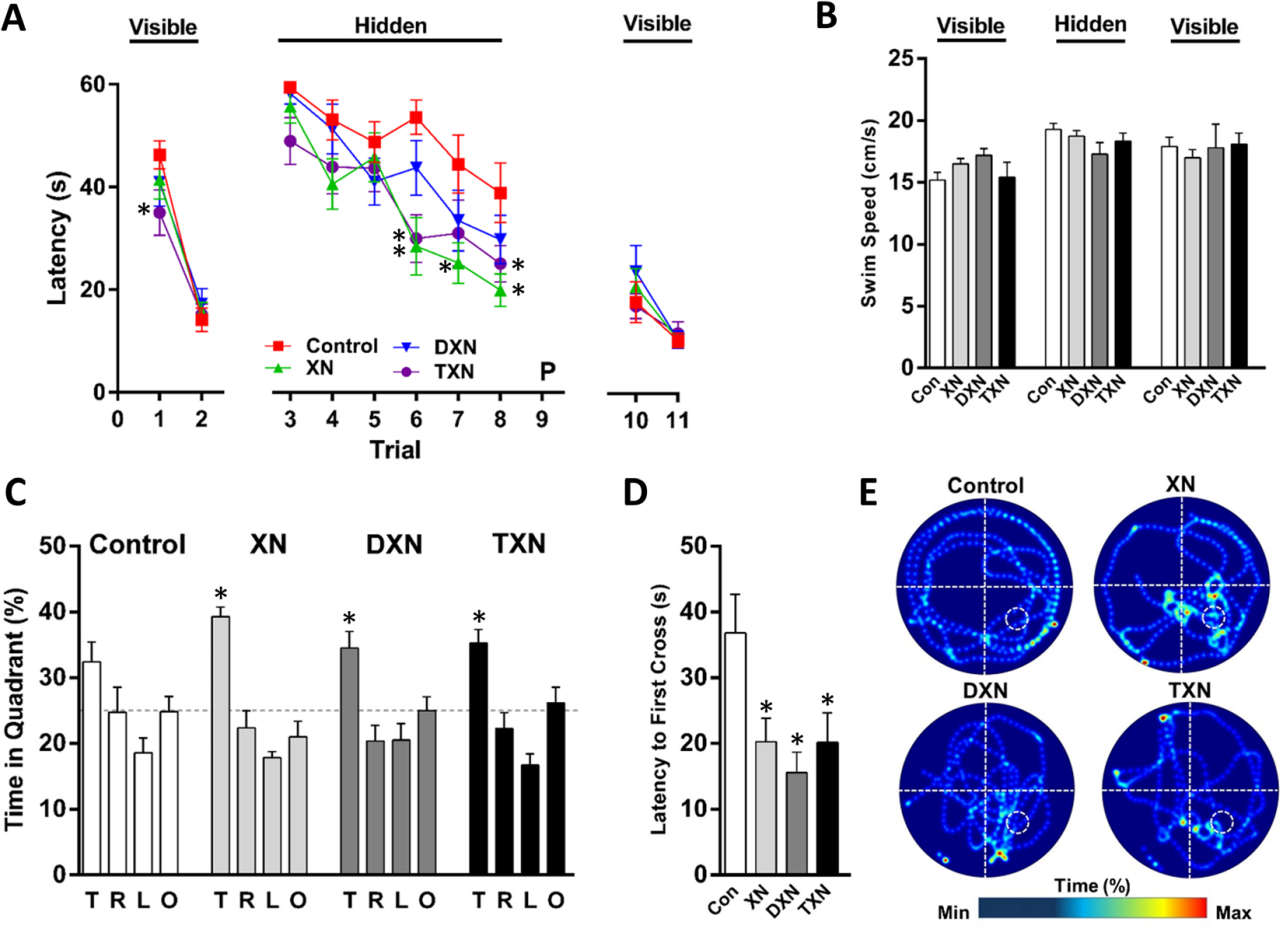
XN and its derivatives improve spatial learning and memory in HFD-fed mice. **(A**) Learning curves in visible and hidden platform trials of the water maze. While there were no treatment effects on ability of the mice to locate the visible platform, treatment effects were seen when the platform was hidden. Mice treated with XN or TXN showed better performance than control mice. *p < 0.05 compared to vehicle, repeated measures ANOVA. P: probe trial (see **C**). (**B**) There were no effects of treatment on the mean swim speed (cm/s) during the first two visible platform trials. Swim speed was calculated during the water maze using a video tracking system. (**C**) Long term spatial memory retention was tested in a 60-s probe trial (P, no platform) 24 h following the completion of the final hidden platform trial. Time spent in the Target (T), Right (R), Left (L) and Opposite (O) quadrants during the 24-h memory probe trial is shown in panel C. The dotted line marks the expected percent time in each quadrant due to chance (25%). *p < 0.05 compared to time in spent in the R, L, and O quadrants, ANOVA, followed by Dunnett’s multiple comparisons test. (**D**) Latency to first crossing of the platform position during the 24-h memory probe trial. *p < 0.05 compared to control group, ANOVA. (**E**) Representative heat maps show the swim patterns of mice during the memory probe trial. The platform location is marked by a white circle (bottom right, Target quadrant). **A**–**D**, n = 11–12 mice/treatment.

To determine the effects of XN, DXN, and TXN on spatial learning and memory and long-term memory retention, we tested mice in the water maze (Fig. 9). Mice were first trained to locate a visible platform in two trials (Fig. 9A, left), in which an escape platform was clearly marked with a visual cue. On the first trial, mice fed TXN reached the platform 24% faster compared with mice fed control, whereas no differences were observed in the second trial. There were no differences in swim speeds among groups (Fig. 9B).

Spatial learning was assessed during hidden platform trials, in which mice must remember and locate the position of a submerged escape platform using extra maze cues (Fig. 9A, center). On the first hidden platform trial (trial #3 in Fig. 9A), mice fed TXN reached the platform 18% faster compared with mice fed only the HFD, similar to what we had observed with the visible platform. Group differences were significant again for trials 6–8, as mice fed only the HFD improved slower than mice fed XN, DXN, and TXN. Mice fed XN reached the platform faster in trials 6 (47% faster), 7 (43% faster) and 8 (49% faster) compared with mice fed only a HFD. Mice fed TXN reached the platform faster in trials 6 (44% faster) and 8 (35% faster) compared with mice fed only a HFD. When averaging the results of trials 6 to 8, mice fed XN (54% faster), DXN (28% faster), and TXN (45% faster) reached the platform faster than mice fed only HFD. The smaller differences between control and DXN in reaching the platform can be explained in part by the 10% slower swim speed in mice fed DXN versus mice fed only HFD during the hidden platform trials. Another way to show how control-fed mice had impaired spatial learning is to count the number of mice that reached the hidden platform at least twice within 50 seconds. For trials 6 to 8, only 3 out of 12 control-fed mice reached the hidden platform at least twice within 50 seconds, whereas all XN- and TXN-fed mice (both *p* = 0.0003 compared with control-fed mice) and 9 out of 12 DXN-fed mice (*p* = 0.04) were able to achieve this goal.

To test spatial memory retention, mice were administered a probe trial (no platform) 24 h following the completion of the last hidden platform trial. Mice fed XN (45% faster), DXN (58% faster), and TXN (45% faster) reached the platform location faster than mice fed control (see latency to first cross in Fig. 9D). Another way to show how control-fed mice had impaired spatial memory retention is to count number of mice that reached the platform location within 35 seconds. Only 5 out of 12 control-fed mice reached the platform location within 35 seconds, whereas all but one XN-, DXN-, and TXN-fed mice (all *p* = 0.04 compared with control-fed mice) achieved this goal. As shown in the representative heat maps (Fig. 9E) and measured by times spent in the Target quadrant (T, Fig. 9E), XN-fed, but not DXN- and TXN-fed, mice spent 6.8% more time in the correct quadrant than control-fed mice (Fig. 9C). Another way to show how control-fed mice had an impaired spatial memory retention is to determine the number of mice that spent more time in the correct quadrant than in any other quadrant. Only 3 out of 12 control-fed mice spent most of the time in the correct quadrant, whereas all but one XN- and TXN-fed mice (both *p* = 0.003 compared with control-fed mice) and 9 out of 12 DXN-fed mice (*p* = 0.04) achieved this goal.

To reconfirm that these effects were specific to spatial learning and memory rather than differences in motivation, mice were administered two additional visible platform trials following completion of the probe trial. There were no significant differences amongst the groups in these final visible platform trials (Fig. 9A, right). Thus, XN and its derivatives ameliorated HFD-induced impairments in spatial learning and memory.

Altered measures of anxiety can affect performance in the water maze; therefore, we assessed thigmotaxis in the mice. There were no differences in thigmotaxis between groups during the visible or hidden platform trails or the probe trial (*p* = 0.62, *p* = 0.31, and *p* = 0.97, respectively).

## Discussion

An estimated 35% of US adults met criteria for MetS in 2012, significantly increasing their risk of cardiovascular disease and T2D^22^. MetS is also associated with cognitive impairment^6,55–57^, a significant concern considering the epidemic proportions of MetS and current lack of any agent to treat it effectively. Direct health care costs arising from obesity and/or related disorders account for ~7–10% of annual US health care expenditures^58^, and the need for effective MetS therapies is pressing^59^. Our group has previously explored the efficacy of XN in preclinical models of MetS^32,36,60^. Despite encouraging results, concerns have been raised regarding the estrogenicity of XN’s gut microbial metabolite, 8-PN^45,46,48^, which poses a potential health risk for its use in humans. Possemiers and co-workers^48^ have argued that the existence of 8-PN producing gut microbial metabotypes in the human population might elicit estrogenic effects in humans consuming moderate amounts of beer. While microbrew beers may contain up to 4 mg/l of combined XN and IX^61^, human exposure to XN and 8-PN through consumption of supplements containing XN-enriched extract may be much higher. In the current study, we assessed the biological activity of two hydrogenated XN derivatives, DXN and TXN, that cannot be converted to 8-PN. Our findings show that XN, DXN and TXN have very low affinity for the estrogen receptors compared to 8-PN (Table 1). In addition, they do not stimulate proliferation or induce PGR expression in MCF-7 breast cancer cells, suggesting that they lack classic estrogenic activity (Figs 2 and 3). The two hydrogenated compounds also gave rise to higher steady-state levels than XN. Furthermore, DXN and TXN improved markers of peripheral metabolism in HFD-fed mice without inducing liver toxicity, as indicated by plasma AST and ALT levels (Table 2) and by cell viability assays with HepG2 cells (Supplementary Fig. S1b), and they ameliorated HFD-induced impairments in hippocampus-dependent learning and memory. Thus, the XN derivatives DXN and TXN represent potential compounds for the treatment of the neuro-metabolic impairments associated with HFD-induced obesity, insulin resistance and MetS.

DXN and TXN treatment resulted in lowering of leptin levels compared to the control fed mice. Leptin enters the vascular circulation in proportion to fat mass^62^ and following its saturable transport across the blood brain barrier into the hypothalamus, plays an important role in regulation of appetite and energy expenditure^63^. Obesity-induced hyperleptinemia typically coexists with loss of responsiveness to leptin and may contribute to MetS with peripheral leptin acting as a potent proinflammatory cytokine^64,65^. In addition, convergence of leptin and insulin intracellular signaling in the hypothalamus to regulate energy metabolism may be impeded by development of either leptin or insulin resistance^64,65^. The lower leptin levels in mice treated with DXN and TXN are likely due to a reduction in fat accumulation and may contribute to the improved metabolic profile by maintaining optimal leptin signaling and reducing inflammation.

Of the three compounds tested, only TXN was found to reduce body mass gain, feed efficiency, and fasting plasma glucose levels (Table 2). The data suggest that TXN is more potent than XN or DXN in increasing energy expenditure to explain the significant reduction of body mass gain and feed efficiency by TXN. TXN was most effective in increasing oxygen consumption in C2C12 cells due to mild mitochondrial uncoupling (Fig. 8A), which could be one mechanism for explaining an increase in energy expenditure and decreased body mass gain induced by TXN. Another possible mechanism for the differential effects of TXN, DXN and XN on body mass and feed efficiency is the reduction in the absorption of dietary fats. Some flavonoids such as epigallocatechin-3-gallate are known to inhibit the absorption of dietary fats by inhibiting pancreatic lipase resulting in increased fecal lipid excretion^66^. Further work is needed to explore this mechanism for TXN, although any reduction of fat absorption would not explain the observed mild mitochondrial uncoupling. The reduction of fasting plasma glucose by TXN but not by XN and DXN may be explained by the greater effect of TXN on insulin sensitivity as indicated by the marked decrease in HOMA-IR by TXN (Table 2) and by the results of the glucose tolerance test (Fig. 6A and B) showing TXN having the greatest effect on glucose clearance at the tested dose regimen.

Unlike plasma glucose, plasma triglycerides and total cholesterol were unaffected by TXN treatment (Table 2). This finding is not unexpected as we have previously observed a significant reduction of hepatic triglyceride content by XN, dosed at 30 mg/kg/day, in diet-induced obese mice without a significant change in plasma triglycerides^36^. Feeding polyphenol-rich aqueous extract of Rutgers Scarlet Lettuce also caused a reduction of total liver lipids but not plasma lipids in diet-induced obese C57BL/6 mice^67^. Supplementation with polyphenol-enriched fraction from the Chinese herb, Erigerontis Herba, significantly decreased body mass gain of mice without a significant reduction in plasma total cholesterol and triglycerides^68^. At the tested dose regimen, the major beneficial health effects of TXN on metabolic syndrome appears to be its ability to reduce blood sugar levels and body weight gain without lowering plasma lipid levels.

We have previously shown that XN is a potent inducer of quinone reductase^69^, one of the enzymes induced by activation of the antioxidant Keap1-Nrf2 pathway. XN also effectively inhibits lipid peroxidation of human LDL induced by SIN-1, whereas TXN afforded little or negligible protection^70^ suggesting that the α,β-unsaturated keto functionality of the chalcone structure of XN is important for its antioxidant activity. The α,β-unsaturated keto functionality present in XN may also give this compound greater potency than DXN or TXN for alleviating MetS-associated cognitive impairment (Fig. 9).

XN has been shown to activate AMPK in mouse liver^71,72^, skeletal muscle^72^, lung^73^, mouse embryonic fibroblasts^74^ and human endothelial cells^75^ through the liver kinase B1 pathway in fibroblasts^74^ and the CAMMKβ pathway in endothelial cells^75^. In our study, XN given in the diet at a dose of 30 mg/kg/day to 9-week old C57BL/6J mice for 13 weeks activated AMPK in the liver (Fig. 7A,B), consistent with findings from other groups^71,72^, whereas DXN and TXN treatment had no effect on AMPK activation. Costa and co-workers^72^ observed improvement of MetS parameters following treatment with XN and 8-PN, which they administered to 36-week old male C57BL/6 mice through the drinking water with 0.1% ethanol for 20 weeks. They attributed the improvement of MetS biomarkers to activation of AMPK in liver and skeletal muscle. In our study, XN, DXN, or TXN treatment resulted in approximately 20% attenuation of AMPK phosphorylation in skeletal muscle (Fig. 7C,D). While there is no obvious explanation for the observed difference, the two studies are very different with respect to dose regimen, route of administration, length of treatment, selected endpoints (*e.g*., leptin, cognitive function), and age of the mice. In our study, the improvements in cognitive function and glucose metabolism following treatment with XN, DXN and TXN may not be directly related to AMPK phosphorylation in liver or skeletal muscle. As a case in point, leptin, whose levels decreased in response to XN^36^, DXN and TXN treatments (Table 2), inhibits hypothalamic AMPK but increases AMPK phosphorylation in skeletal muscle^76^. XN-induced leptin downregulation may therefore indirectly downregulate AMPK activation in muscle.

Unlike our previous XN study^36^, the present study showed no reduction in body weight gain following XN treatment at 30 mg/kg/day (Table 2). The previous study and the present study were not carried out under identical conditions. The previous study was a 12-week feeding study whereas the present study lasted 13 weeks. Furthermore, although the same strain and age (9 weeks) of C57BL/6J mice were used in both studies, the starting weights of the experimental mice were different in the two studies (21.6 ± 0.03 g in the previous study vs. 24.1 ± 0.25 g in the present study). The difference in initial body weight might explain the difference in body weight gain over time because fatness has been shown to be the strongest predictor of the variability in weight gain independent of the duration of HFD feeding^77^. This finding suggests that the effects of XN on glucose metabolism do not follow changes in body mass because XN treatment resulted in improved glucose tolerance (Fig. 6A,B).

The effects of XN, DXN and TXN on mitochondrial function were examined using C2C12 myoblasts as a model of cellular energy generation and expenditure. We employed C2C12 cells as an *in vitro* model because of their high bioenergetics activity. In these experiments, we selected a concentration of 5 µM based on previous findings that 5 µM XN caused a significant increase in OCR^60^. Our previous^32^ and present findings (Fig. 5) show that tissue concentrations in the range 0.3–3 µM are attainable with a mouse equivalent dose regime of 30 mg/kg/day which, by allometric interspecies scaling of dose^78^, corresponds to a human equivalent dose of 175 mg/day for a 70-kg person. At 5 µM concentration, XN, DXN and TXN acted as mitochondrial uncouplers in C2C12 cells as shown by their ability to significantly increase the OCR after selective inhibition of ATP synthase by oligomycin. In this cell-based assay, TXN and DXN were more potent than XN, suggesting that the α,β-unsaturated ketone functionality of XN is not required for acting as a mitochondrial uncoupler. DXN and TXN have the advantage over XN that they lack (pro-)estrogenic effects and Michael-type reactivity towards proteins. The mechanism of action of the uncouplers can be a direct effect (by acting as protonophores dissipating the proton gradient) or indirectly by stimulating the uncoupling protein (UCP) expression. However, induction of UCP expression would require time. Therefore, in the current study, the uncoupling effect is unlikely the result of transcriptional regulation because it was observed immediately after addition of the test compounds. We previously reported that XN dissipates of ΔΨ from ATP synthesis in cell culture which we associated with reduced body weight gain and improvement of dysfunctional glucose and lipid metabolism in obese Zucker fa/fa rats^60^.

As shown in Fig. 5, the highest concentration we measured for the test compounds was 5 nmol/g TXN in liver tissue (approximately 5 µM) following a mouse dose of 30 mg/kg/day. We therefore determined whether the test compounds exert cytotoxicity at this concentration in HepG2 cells which we chose as our *in vitro* model to detect hepatotoxicity. XN, DXN and TXN did not display hepatotoxicity in the range 1–25 µM (Supplementary Fig. S1b), suggesting that these compounds may not cause liver damage in mice receiving the test compounds at a dose of 30 mg/kg/day or in humans at an equivalent dose of 175 mg/day for a 70-kg person.

There was a difference in hepatic (micromolar) and skeletal muscle (nanomolar) concentrations of XN and its derivatives (Fig. 5). The difference is due to hepatic extraction of the xenobiotics, also referred to as the ‘first-pass effect’. XN and its hydrogenated derivatives are released from the liver into the general circulation primarily as glucuronide conjugates^79,80^ which are not taken up by peripheral tissues. Only the non-conjugated forms of the test compounds can be taken up by peripheral tissues. This explains the much lower concentrations found in skeletal tissue because the circulating concentrations of non-conjugated XN and its derivatives are <5% compared to the plasma glucuronide concentrations.

Our group has previously shown that XN, at a dietary dose of 30 mg/kg body weight, improves cognitive flexibility in young animals fed a standard diet for 8 weeks^81^. Here, we show that XN and its derivatives, DXN and TXN, improve hippocampus-dependent learning and memory in mice fed a HFD. The improvement in cognitive function in these HFD-fed mice (Fig. 9) was associated with increased glucose tolerance (Fig. 6). Insulin resistance is one of the key components of MetS and may represent as a major link between cognitive impairment and MetS^82–84^. Insulin plays a key role in normal learning and memory, and insulin resistance has been reported to affect cognition^85–87^. Impairment of insulin signaling in brain neurons may cause dysregulation of energy metabolism and impact cell repair and survival, and dysfunctional insulin signaling produces metabolic alterations that contribute to the development of neurodegenerative diseases^88^.

## Conclusion

XN, DXN, and TXN improve glucose tolerance and cognitive function in HFD-fed mice. Unlike XN, DXN and TXN are unable to form the estrogenic metabolite, 8-PN, and they themselves have negligible affinity for estrogen receptors. Taken together, hydrogenation of the α,β-unsaturated keto moiety of XN resulted in greater steady-state concentrations, loss of affinity for the estrogen receptor, and retention or even enhancement of the beneficial effects of XN on HFD-induced dysfunctional glucose metabolism and impaired cognitive function. Thus, the XN derivatives DXN and TXN have potential to prevent or treat the neuro-metabolic impairments associated with HFD-induced obesity and MetS without risk of liver injury and adverse estrogenic effects.

## Methods

### Materials

XN and TXN (both of purity >99%) were provided by Hopsteiner, Inc. (New York, NY, USA). Oleic acid was purchased from TCI America (Portland, OR, USA). Oligomycin and the synthetic uncoupler, FCCP (carbonyl cyanide-4-(trifluoromethoxy)phenylhydrazone), were purchased from Seahorse Bioscience (North Billerica, MA, USA). MTT (3-(4,5-dimethylthiazol-2-yl)-2,5-diphenyltetrazolium bromide) was obtained from Sigma-Aldrich (St. Louis, MO, USA). All other reagents were purchased from Sigma-Aldrich.

### α,β-Dihydroxanthohumol (DXN) and tetrahydroxanthohumol (TXN)

DXN was synthesized from XN by catalytic hydrogenation using Wilkinson’s catalyst (RhCl(PPh_3_)_3_). The reaction mixture consisted of XN, 10 mol% of RhCl(PPh_3_)_3_, ammonium formate (source of hydrogen, 4 eq), and [bmim][BF_4_] ionic liquid (6.3 eq, 8.5 mmol) as a solvent. After stirring the reaction mixture at 90 °C for 3 h, it was extracted with ethyl acetate and the extract washed with water. The organic layer was dried with anhydrous Na_2_SO_4_ and evaporated under reduced pressure. The crude product was purified by flash chromatography on a silica gel column and eluted with ethyl acetate: hexane (1:1.7, v/v). DXN was isolated as a pale yellow solid with a yield of 44%. The compound was characterized by NMR and MS/MS analysis (Purity > 99%, Supplementary Figs S2–S4. TXN, provided by Hopsteiner, Inc. (New York, NY, USA) was characterized by NMR (Supplementary Figs S5 and S6) and MS/MS analysis (purity > 99%, Supplementary Fig. S7).

### Estrogen receptor binding affinity

Relative binding affinities of XN, DXN and TXN for full-length purified human estrogen receptors ERα and ERβ were determined by competitive radiometric binding assays using 2 nM [^3^H]-estradiol as tracer ([2,4,6,7-^3^H]-estra-1,3,5(10)-triene-3,17β-diol, 70–115 Ci/mmol, Perkin Elmer, Waltham, MA, USA) as described^89,90^. The receptors and ligands were incubated for 18–24 h at 0 °C and then the receptor–ligand complexes separated using hydroxyapatite (BioRad, Hercules, CA, USA). Binding affinities were expressed as relative binding affinity values, with the affinity of estradiol for ERα or ERβ set at 100%.

### MCF-7 Cell Proliferation using sulforhodamine B (SRB) assay

The SRB assay has been used to study the effects of phytochemicals such as XN, DXN and 8-PN on cell proliferation of various cancer cell lines, including MCF-7 cells^91,92^. SRB is a dye that binds to cellular proteins of adherent cells and the absorbance readings are a measure of the number of viable cells (cell mass) attached to the culture plate after incubation with the test compounds (samples) in comparison with vehicle control cells. The cell mass is then extrapolated as a measurement of cell viability and cell proliferation^93^. Increased proliferation of MCF-7 cells is one of the measures of estrogenicity of phytochemicals^94^.

MCF-7 cells were plated at 10,000 cells/well in 96-well plates in DMEM medium containing 10% charcoal-stripped FBS and antibiotics. The cells were treated at different concentrations (0.001, 0.01, 0.1, 1, and 10 μM) with XN, DXN, TXN, and 8-PN . Positive control cells were treated with 10 nM 17β-estradiol (E2) whereas controls (vehicle alone) were treated with ethanol not to exceed 0.1%. After incubation with the test compounds for 48 h, the cell culture medium was removed from each well of the 96-well plate and the cells were fixed for 1 h with 10% trichloroacetic acid. The cells on the plate were washed five with distilled water, air-dried, and then stained with SRB dye (0.4% w/v in 1% acetic acid). Excess dye was removed by washing the plate five times with 1% acetic acid. The plates were air-dried and the adsorbed dye was solubilized in 10 mM Tris buffer for 5 min on a shaker before reading the optical density (OD) at 565 nm. The percentage cell viability/proliferation was calculated as follows:

$$({{\rm{OD}}}_{{\rm{Sample}}}-{{\rm{OD}}}_{{\rm{Zero}}{\rm{blank}}})/({{\rm{OD}}}_{{\rm{Control}}}-{{\rm{OD}}}_{{\rm{Zeroblank}}})\times 100.$$

Mean values and the coefficient of variation (CV) from six replicate wells were calculated and a one-way ANOVA was used to compare statistical differences between treated and control cells, with *p* < 0.05 as the level of significance.

### RNA isolation and real-time PCR

MCF-7 cells (American Type Culture Collection, Manassas, VA, USA) were cultured in DMEM (Corning Life Sciences, Tewksbury, MA, USA) supplemented with 10% fetal bovine serum (Corning Life Sciences) and 100 μg/ml penicillin/streptomycin (Invitrogen, Carlsbad, CA, USA). For estrogen-free experiments, cells were seeded in phenol-free DMEM (Gibco/Life Technologies, Grand Island, NY, USA) plus 10% charcoal-dextran-treated FBS (Corning Life Sciences) at 5 × 10^5^ cells/well of a 6-well plate. Cells were treated with XN, DXN, TXN, 8-PN (0.2, 1 and 5 μM) and 17β-estradiol (1 and 10 nM) for 20 h. Total RNA was isolated using TRIzol (Invitrogen) and converted into cDNA using iScript (Bio-Rad, Hercules, CA, USA). Real-time PCR was performed in triplicate on a CFX-96 (Bio-Rad) using primer-probe assays (IDT Technologies, Coralville, IA, USA) and SsoAdvanced Universal Probes Supermix (Bio-Rad) to quantify progesterone receptor (PGR) and GAPDH levels. The ratio of PGR to GAPDH was calculated as 2^−ΔCt PGR^/2^−ΔCt GAPDH^. Data were analyzed using a one-way ANOVA (*p* < 0.05 was considered statistically significant) and a post-hoc Tukey’s multiple comparison test. Induction of the progesterone receptor in MCF-7 cells is one of the indices of estrogenicity of flavonoids such as 8-PN^94^.

### Animal studies and diets

Nine-week old male C57BL/6J mice, obtained from The Jackson Laboratory (Bar Harbor, ME, USA), were housed individually in plastic cages under a 12–12-hr light-dark cycle. They were assigned randomly to four groups consisting of 12 mice each. One group (control) was fed a HFD (Dyets Inc., Bethlehem, PA, USA) containing 60%, 20% and 20% total calories from fat, carbohydrate and protein, respectively, and the other three groups were fed the HFD containing XN, DXN, or TXN. The test compounds were dissolved in an isotropic mixture of oleic acid:propylene glycol:Tween 80, 0.9:1:1 by weight (OPT) before incorporation into the HFD. The control diet contained an identical amount of OPT. All diets were prepared in pellet form by Dyets, Inc. (Bethlehem, PA, USA). The mouse diets were formulated to deliver a dose of 30 mg test compound per kg body weight per day. Food intake was measured three times per week and body weights were recorded weekly. At the end of 13 weeks of feeding, the mice were euthanized and blood, liver and skeletal muscle were collected for analyses. All animal protocols were approved by Institutional Animal Care and Use Committee (IACUC) at Oregon State University. Animal experiments were carried out in accordance with the relevant guidelines and regulations.

### Metabolic and liver toxicity measurements

Plasma triglycerides and total cholesterol were analyzed with commercially available kits (Fisher-Scientific, Pittsburgh, PA, USA). Plasma glucose was analyzed by a test kit obtained from Wako Diagnostics (Mountain View, CA, USA). Plasma insulin and leptin were analyzed by ELISA kits purchased from Alpco Diagnostics (Salem, NH, USA) and Crystal Chem (Downers Grove, IL, USA), respectively. Homeostasis Model Assessment of Insulin Resistance (HOMA-IR) was calculated as follows: fasting plasma insulin concentration (mU/L) × fasting plasma glucose level (mmol/L)/22.5^95^. Plasma levels of AST and ALT were measured using commercially-available kits (Bioo Scientific Corp., Austin, TX, USA) as indicators of *in vivo* liver toxicity.

### LC-MS Analysis of XN, DXN and TXN in mouse liver, plasma, and muscle

Liver and plasma from each mouse were subjected to enzymatic hydrolysis, extracted and analyzed using the paper strip extraction technique as previously described^36,96^. For skeletal muscle, approximately 200 mg of frozen muscle tissue were placed into tubes containing ceramic beads (1.4 mm Ceramic, Bulk Beads, Fisher Scientific, Pittsburgh, PA, USA) and methanol:water (90:10; 500 μL/200 mg tissue) and homogenized using a Precellys 24 (PEQLAB Biotechnology GmbH, Germany). The tissue was homogenized for 3 × 30 sec at 6,500 rpm with 20 sec intervals and then centrifuged at 15,700 × *g* for 10 minutes and 100 μl of supernatant was used for the paper strip extraction as previously described^36,96^. Concentrations of XN, DXN and TXN were calculated using an internal standard, 2′,4-dihydroxychalcone (DHC), with matrix-based calibration curves and Analyst Software (Analyst 1.5, AB Sciex, Foster City, CA, USA). Samples were analyzed by LC-MS/MS using an AB Sciex 4000 Q-trap triple quadrupole mass spectrometer using a source temperature of 600 °C and a needle voltage of −4500 V. The source, curtain and collision gases were all nitrogen. Analytes were separated by high performance liquid chromatography (HPLC) using a 2.1 × 50 mm Agilent Zorbax 300 SB – C8 3.5 µm column (Agilent, Santa Clara, CA, USA). The elution gradient was 25 to 60% solvent B (0.1% formic acid in acetonitrile) in solvent A (aqueous 0.1% formic acid) in 2.6 minutes using a flow rate of 0.5 ml/min after an initial 1.4 min at 25% solvent B. The gradient was then increased to 100% solvent B in 0.1 min and held at 100% B for 1.4 min, then dropped to 25% B in 0.25 minutes, followed by re-equilibration with 25% B for 9.25 minutes. SRM transitions for quantification were: m/z 353 → 119 for XN, m/z 353 → 233 for IX, m/z 339 → 219 for 8-PN, m/z 355 → 249 for DXN, m/z 357 → 251 for TXN, and m/z 239 → 119 for DHC.

### Glucose tolerance

Glucose tolerance tests were conducted after 4 and 11 weeks of feeding the experimental diets. Five mice from each treatment group were used in each glucose tolerance assay. Mice were fasted for 6 h prior to baseline blood glucose testing. Blood was collected from the mice by tail puncture, and glucose measurements were taken using a One Touch UltraMini glucometer (LifeScan, Inc., Milpitas, CA, USA). Mice were weighed and then given an i.p. injection of glucose equal to 2 g/kg body weight. Glucose readings were taken at 0 minutes (prior to glucose injection), and at 15 min, 30 min, 1 h, and 2 h after glucose injection.

### AMPK activation by Western blot analyses of liver and skeletal muscle proteins

Approximately 150 mg of frozen liver and muscle tissues were homogenized in tubes containing RIPA buffer with protease and phosphatase inhibitors plus ceramic beads (1.4 mm Ceramic, Bulk Beads, Fisher Scientific, Pittsburgh, PA, USA) using a Precellys 24 (PEQLAB Biotechnology GmbH, Germany). Liver tissue was homogenized for 1 × 30 sec at 6,500 rpm whereas muscle was homogenized for 3 × 30 sec at 6,500 rpm with 20 sec intervals. The homogenates were centrifuged at 15,700 × *g* for 10 minutes and aliquots (50 µg of protein) of the supernatant (lysate) were separated by SDS-PAGE using 4–15% MP TGX Gels (Bio-Rad # 4561083) and blotted onto nitrocellulose membranes. Membranes were blocked with 5% nonfat milk for 2 h and incubated with antibodies against pAMPK (Phospho-AMPKα (Thr172) Antibody, cat. no. #2531 Cell Signaling Technology, Danvers, MA, USA), AMPK (AMPKα Antibody, cat. no. #2532 Cell Signaling Technology, Danvers, MA, USA), or β-actin (Santa Cruz Biotechnology) for 1 h or overnight. After a second reaction with secondary antibodies (horseradish peroxidase-conjugated IgG goat antirabbit or goat antimouse antibodies), the protein bands on the nitrocellulose membranes were visualized by enhanced chemiluminescence substrate on a Biorad ChemiDocTM MP Imaging System (BioRad Laboratories Inc, Hercules, CA, USA). Band intensities were quantified by densitometry using Image J software.

### MTT assay

For cell viability experiments using the MTT assay, mouse C2C12 skeletal muscle myoblasts (ATCC; Manassas, VA, USA) and the human hepatocyte cell line, HepG2 (ATCC; Manassas, VA, USA), were plated in 96-well plates at a density of 4,000 to 10,000 cells per well in 200 μL of DMEM medium supplemented with 10% FBS, 1% glutamine, 1 mM of sodium pyruvate, 100 units/mL penicillin, and 100 μg/mL streptomycin. After incubating 48 h, the medium was removed and the cells were treated with fresh solutions of various concentrations of XN, DXN or TXN in phenol red-free DMEM plus supplements. The final concentrations of XN, DXN, TXN were 1, 2, 5, 8, 10, 25, or 50 μM, in quadruplicate wells. The plates were then incubated for 1 h (for C2C12) or 24 h for HepG2. After removing the medium, the cells were incubated for 2 h with a 0.5 mg/mL solution of MTT in phenol red-free culture medium. After removing the medium and adding a solution of acidified isopropanol to each well, the absorbance of each well was measured at 570 nm by using a spectrometer (SpectraMax 190, Molecular Devices, Sunnyvale, CA, USA). Cell viability of compound-treated cells was calculated as % absorbance of vehicle-treated control cells (Supplementary Fig. S1a and S1b).

### Oxygen Consumption Rate (OCR) and Extracellular Acidification Rate (ECAR) measurements

OCR and ECAR measurements were performed with a Seahorse XF 24 Analyzer (Agilent Technologies, Inc., Santa Clara, CA, USA). C2C12 cells were plated in culture medium at a density of 40,000 cells per well in a 24-well plate. The cells were then allowed to adhere for 24 h (37 °C, 5% CO_2_). Prior to the assay, the cells were washed with freshly prepared running medium consisting of Seahorse XF Assay medium (Agilent Technologies, Inc., Santa Clara, CA, USA), 10 mM glucose and 1 mM sodium pyruvate. The pH of the medium was adjusted to 7.4 with 1 M NaOH. The cells were then equilibrated for one hour at 37 °C without CO_2_. The ECAR measurements are highly sensitive to fetal bovine serum (FBS) in growth medium and it is suggested that little or none be present. XN, DXN, and TXN are highly nonpolar and previous experiments suggested FBS should be present in media to dissolve XN. To overcome this problem, the test compounds were first dissolved in ethanol and the resulting solution was diluted 100-fold in treatment medium (9 parts XF media and 1 part 10% FBS media) which was added directly to cells. The treatment medium contained 1% FBS and after a further 10-fold dilution into the media in wells, only 0.1% FBS was present during OCR and ECAR measurements. The final concentrations in the wells were: 1 μM for oligomycin, and 5 μM for XN, DXN or TXN. The concentration of oligomycin and the cell density used were selected based on preliminary experiments. Ethanol was added to the control wells not to exceed a final concentration of 0.1% (vehicle control). OCR and ECAR were measured at 9-min intervals.

### Cognitive function

After 13 weeks of feeding the experimental diets, general task learning, spatial learning and memory, and long-term spatial memory retention were tested using the water maze, as previously described^97,98^. Briefly, the water maze consisted of a circular pool, filled with water (24 °C) made opaque white with non-toxic tempera paint (Prang, Dixon Ticonderoga Company, Heathrow, FL, USA). Spatial cues consisted of figures of geometric shape and other items such as toys and pieces of cloth. The cues were placed on the walls of both the room and the sides of the tank and were present throughout all water maze trials. All trials consisted of a maximum of 60 s in the water searching for the platform and 10 s on the platform. If a mouse failed to find the platform within the designated 60 s swim time, it was led to the platform by the experimenter. Trials on the same day were separated by 10 min. Mice were first trained to locate a visible escape platform (plexiglass circle, 12 cm diameter) submerged 2 cm below the surface of the water, by the use of a cue (a colored cylinder, 2.5 cm radius, 8 cm height) during the visible platform trials (1–2). The location of the platform during visible platform trials was moved between trials to avoid procedural biases in task learning to a specific quadrant. Subsequent to the visible platform training, mice were trained to locate the platform without a visible cue during the hidden platform trials (3–8), which required the mice to rely on extra-maze cues for spatial reference and orientation. To analyze learning curves, time to reach the platform (latency) and swim speeds were assessed. Spatial memory retention was assessed 24 h following the conclusion of the eighth trial of hidden platform training in the probe trial (no platform). For data analysis, the pool was divided conceptually into four quadrants. Time spent searching in the target quadrant compared to the time spent in the three non-target quadrants and the latency to the first crossing of the platform location were analyzed. Following the probe trial, mice were administered two additional visible platform trials. Thigmotaxis, a tendency to remain close the walls, is commonly used as an index of anxiety in mice. For thigmotaxis, we analyzed the time (%) mice spent swimming in the outer part of the pool (<10 cm from edge of pool) during the visible trials. Water maze performance was tracked and analyzed using Ethovision software set at 6 samples/s (Noldus Information Technology, Leesburg, VA). Heat map images were generated using EthoVision Heatmap Generator software and representative images were selected from mice closest to the group mean values for time spent in the quadrant target.

### Statistical analysis

The data were analyzed using SAS 9.2 software (SAS Institute Inc., Cary, NC) and GraphPad Prism 5.0 (San Diego, CA).

Body weight, food intake, and metabolic measurements: data were analyzed using a one-way ANOVA in PROC GLM for single measures within animals (i.e., liver, plasma, and muscle metabolic measures) and a repeated-measures-in-time design ANOVA in PROC MIXED for repeated measures within animals (i.e., body weight, feed intake, and feed efficiency). The area-under-the-curve (AUC) for the glucose tolerance tests were calculated using the trapezoidal rule. Before statistical analysis, data were examined for variance homogeneity and normality. To achieve normality, liver, plasma, and muscle XN data and plasma insulin data were natural log-transformed. For statistical comparisons, the control group was compared with each XN metabolite group.

Water maze learning curves: data, except for spatial learning, were analyzed using a one-way ANOVA for single measures within animals and a repeated-measures-in-time design ANOVA for repeated measures within animals. To evaluate spatial learning, we first compared at each session the control versus each XN metabolite group. For further analysis of spatial learning, the first 3 sessions were used for training of spatial learning and the last 3 session for spatial learning. Because not all mice were able to find the hidden platform within a given trial or within 50 seconds of the trial, we transformed the data into binary values (0 = did not reach platform within 50 seconds; 1 = reached platform within 50 seconds). To show spatial learning, mice had to reach the platform during the last 3 evaluation sessions at least twice within 50 seconds. Fisher’s exact test was used to compare control versus each XN metabolite group.

Seahorse data: to show a consistent baseline, OCR values (in pmol/min/mg protein) and ECAR values (in mpH/min/mg protein) for each value were divided, prior to statistical analysis, by the average of the four pre oligomycin treatment values (0, 8, 17, and 25 min) for the corresponding well and are shown as % pre oligomycin values. To account for repeated measures within wells over time, we utilized a repeated-measures-in-time design using ANOVA procedures in PROC MIXED (SAS). Fixed effects in the model were treatment (control, 5 μM XN, 5 μM DXN, and 5 μM TXN for the compound study), time (0, 8, 17, 25, 34, 43, 52, 60, 69, 78, 86, and 95 min), and their interaction. The random effect was the experiment (5 experiments for the compound study). To account for repeated measures within wells over time, a first order homogeneous variance-covariance matrix [AR(1)] was fitted for each well. Using the ESTIMATE statement, a priori contrasts were constructed by comparing the changes in compound treated cells from the last two time points prior to compound treatment (average 52 and 60 min) to the changes in control cells during the same time-period. This was carried out for each compound and dosage for 69, 78, 86, and 95 min separately and for all four time points combined. Results are shown in the text and the graphs as least squares means and their corresponding SEM (standard error of mean). A *p* value < 0.05 was considered statistically significant.

### Materials and data availability statement

The authors declare that materials, data, and associated protocols will be made available to readers upon request.

## Electronic supplementary material

Supplementary Information
