# Supplementary material for: Non-estrogenic Xanthohumol Derivatives Mitigate Insulin Resistance and Cognitive Impairment in High-Fat Diet-induced Obese Mice
Source: Sci Rep. 2018 Jan 12;8:613. doi: 10.1038/s41598-017-18992-6 (PMC5766630; doi:10.1038/s41598-017-18992-6)

Non-estrogenic Xanthohumol Derivatives Mitigate Insulin Resistance and Cognitive Impairment in High-Fat Diet-induced Obese Mice

Cristobal L. Miranda[1,2], Lance A. Johnson[3], Oriane de Montgolfier[1,2], Valerie D. Elias[1], Lea S. Ullrich[1], Joshua J. Hay[1], Ines L. Paraiso[1,2], Jaewoo Choi[1], Ralph L. Reed[1,2], Johana Revel[1,2,8], Chrissa Kioussi[2], Gerd Bobe[4], Urszula T. Iwaniec[5], Russell T. Turner[5], Benita S. Katzenellenbogen[6], John A. Katzenellenbogen[7], Paul R. Blakemore[8], Adrian F. Gombart[1,9], Claudia S. Maier[8], Jacob Raber[2,3,10*] & Jan F. Stevens[1,2*]

[1]Linus Pauling Institute, [2]Department of [2]Pharmaceutical Sciences, Oregon State University, Corvallis, OR 97331, USA
[3]Department of Behavioral Neuroscience, Oregon Health & Science University, Portland, OR 97239, USA
[4]Department of Animal & Rangeland Sciences, Oregon State University, Corvallis, OR 97331, USA
[5]Skeletal Biology Laboratory, School of Biological and Population Health Sciences, College of Public Health and Human Sciences, Oregon State University, Corvallis, OR 97331, USA
[6]Departments of Molecular & Integrative Physiology, [7]Department of Chemistry, University of Illinois at Urbana-Champaign, Urbana, IL 61801, USA
[8]Department of Chemistry, [9]Department of Biochemistry & Biophysics, Oregon State University, Corvallis, OR 97331, USA
[10]Departments of Neurology and Radiation Medicine, Division of Neuroscience, Oregon National Primate Research Center, Oregon Health & Science University, Beaverton, OR 97006, USA
*Correspondence: fred.stevens@oregonstate.edu (J.F.S.), raberj@ohsu.edu (J.R.)

Supplementary Information: Effect of xanthohumol (XN), α,β-dihydroxanthohumol (DXN), or tetrahydroxanthohumol (TXN) on the viability of C2C12 cells (Figure S1a) and of HepG2 cells (Figure S1b); Structural characterization of DXN by ^{1}H-NMR (Figure S2) and ^{13}C-NMR spectroscopy (Figure S3) and by electrospray mass spectrometry (Figure S4); Structural characterization of TXN by ^{1}H-NMR (Figure S5) and ^{13}C-NMR spectroscopy (Figure S6) and by electrospray mass spectrometry (Figure S7); The MS/MS spectra provided in Figures S4 and S7 were recorded on a Sciex 4000 QTrap mass spectrometer (see Methods). Supplementary Figure S8 contains full-length Western blots of liver and muscle biopsies from untreated mice (control, lanes 1-2) and treated with XN (lanes 3-4), DXN (lanes 5-6) and TXN (lanes 7-8). Tissue extracts were incubated with specific antibodies against pAMPK, AMPK and ß-actin. These original images were used to generate Figure 7. No exposure modifications were recorded.

Figure S1a. Exposure of C2C12 cells to XN, DXN, or TXN for one hour at concentrations 1-50 µM did not affect viability ($p > 0.05$ by ANOVA). Cell viability of treated cells is displayed as a percentage of vehicle treated cells (controls). Data are represented as mean ± SE of 4 replicate wells.

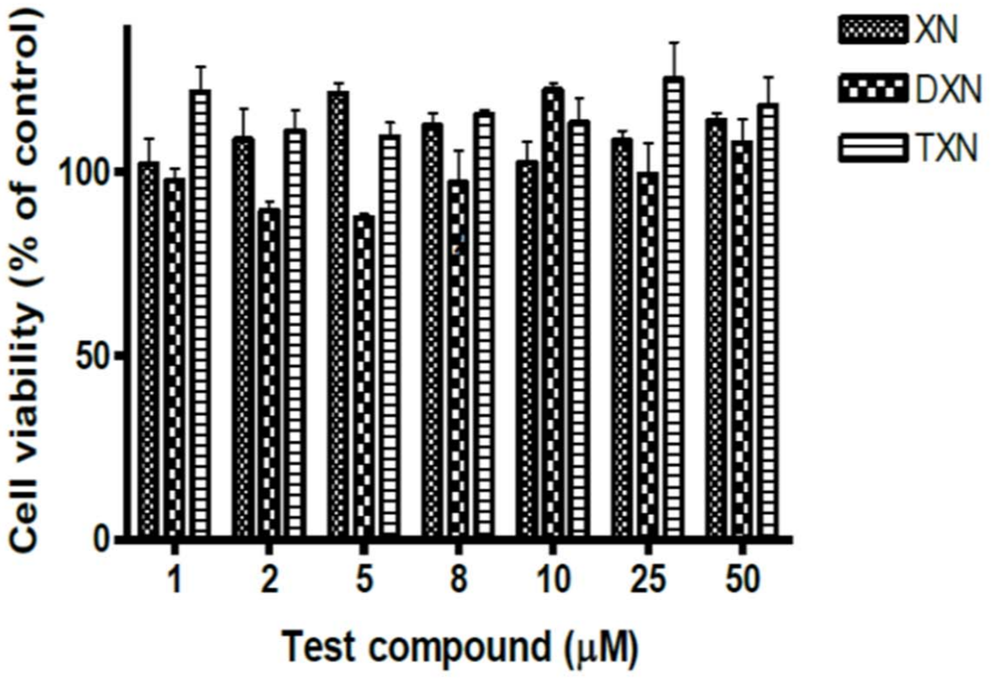

Figure S1b. Exposure of HepG2 cells to XN, DXN, or TXN for 24 h did not affect viability at concentrations up to 25 µM ($p > 0.05$ by ANOVA). However, at 50 µM, all the test compounds were cytotoxic. Cell viability of treated cells is displayed as a percentage of vehicle treated cells (controls). Data are represented as mean ± SE of 4 replicate wells.

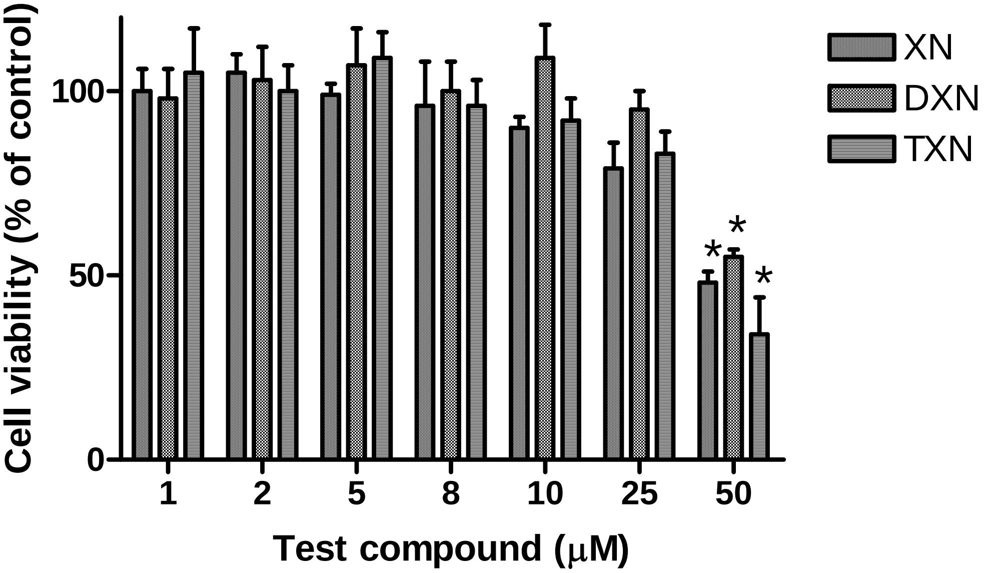

Figure S2. ^{1}H-NMR (700 MHz) spectrum of α,β-dihydroxanthohumol (DXN) recorded in methanol-d_4.

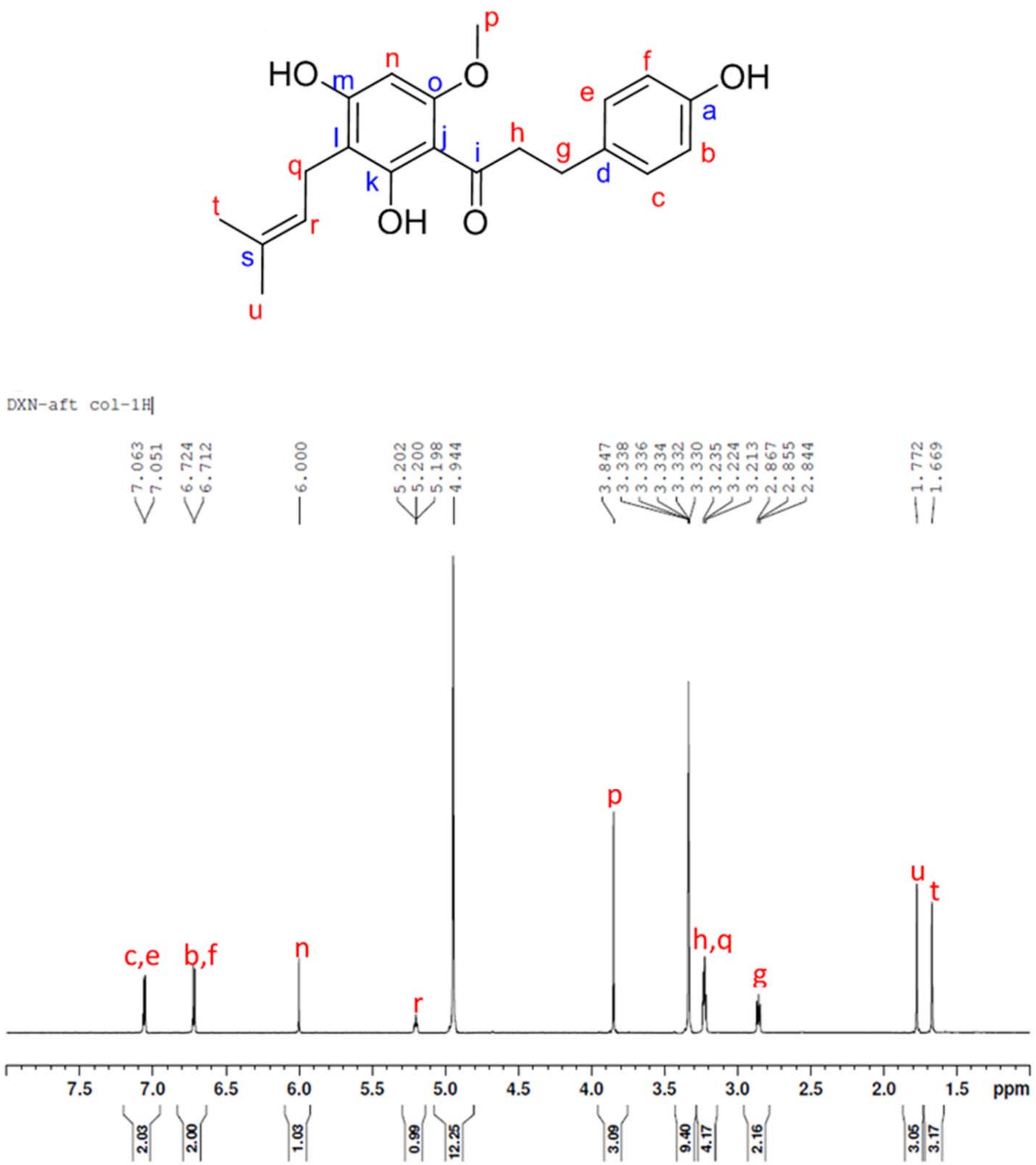

Figure S3. ^{13}C-NMR (175 MHz) spectrum of α,β-dihydroxanthohumol (DXN) recorded in methanol-*d*$_4$. For atom labels, see Figure S2. ^{13}C signals were assigned on the basis of HSQC and HMBC experiments.

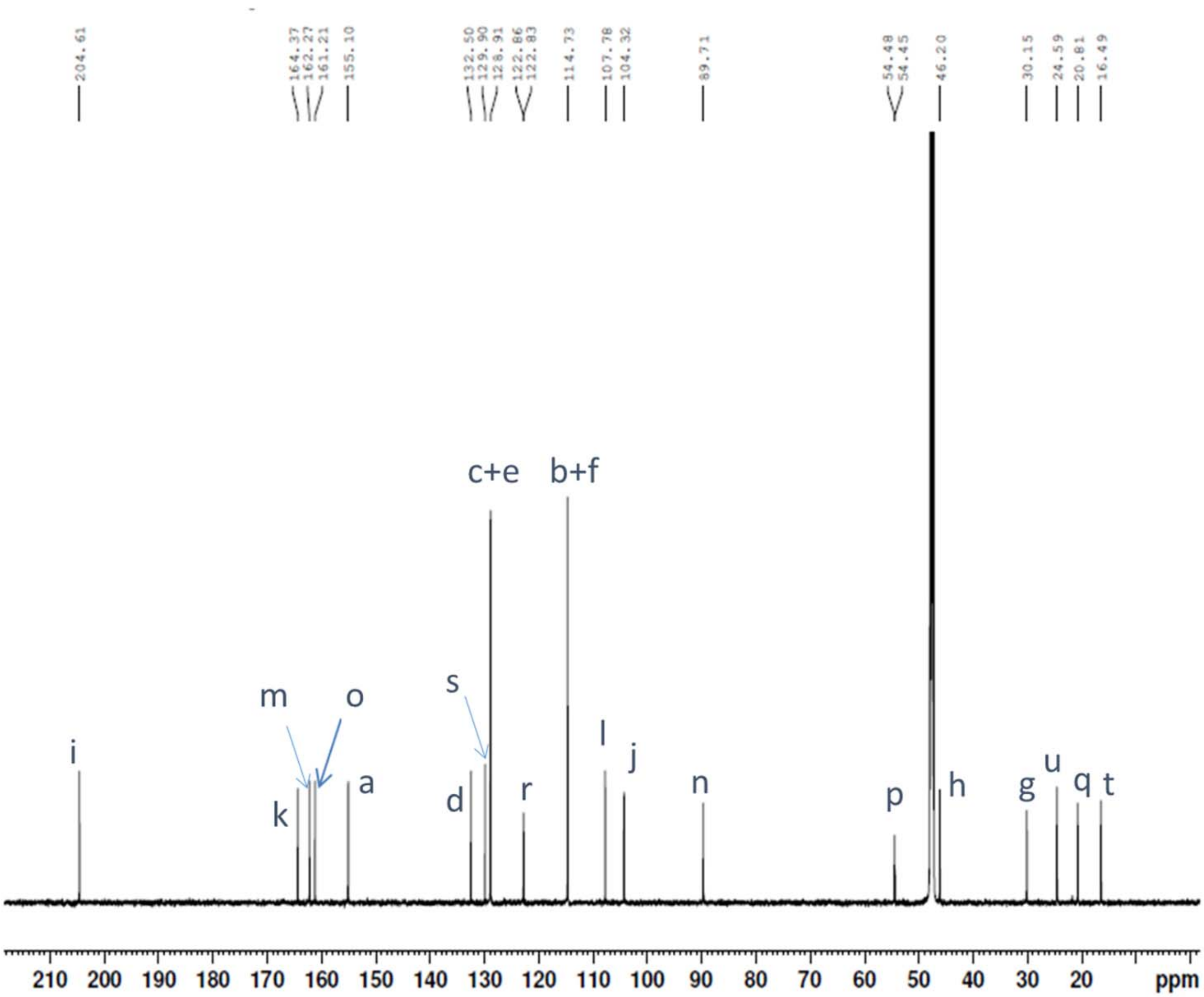

Figure S4. Electrospray MS/MS spectrum of α,β-dihydroxanthohumol (DXN) and its proposed fragmentation pathway.

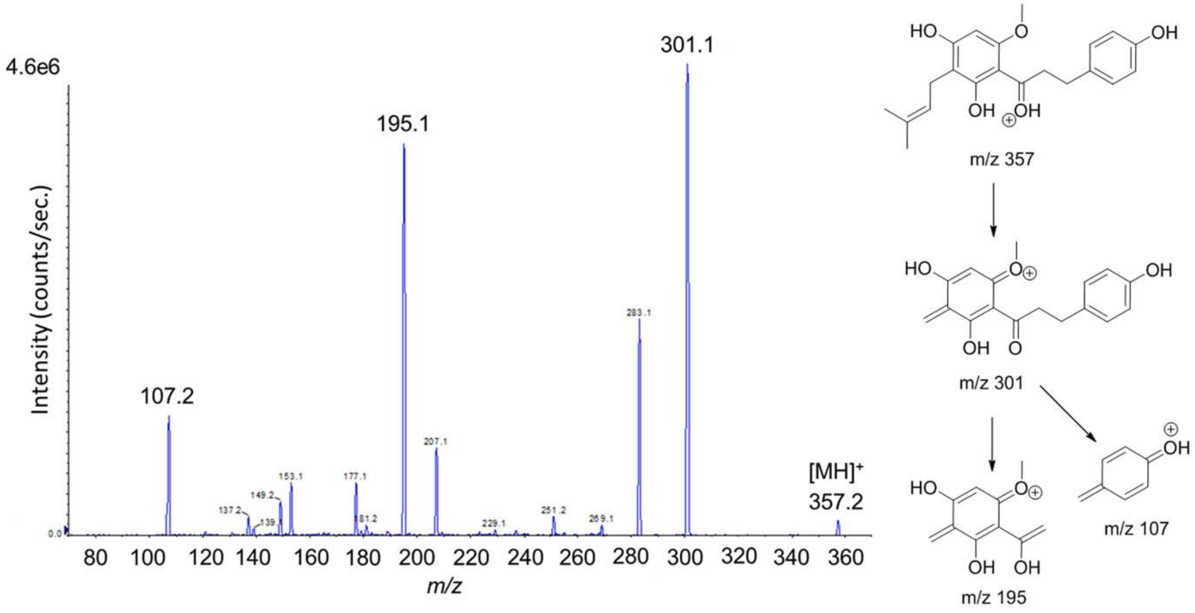

Figure S5. ^{1}H-NMR (700 MHz) spectrum of tetrahydroxanthohumol (TXN) recorded in methanol-d_4.

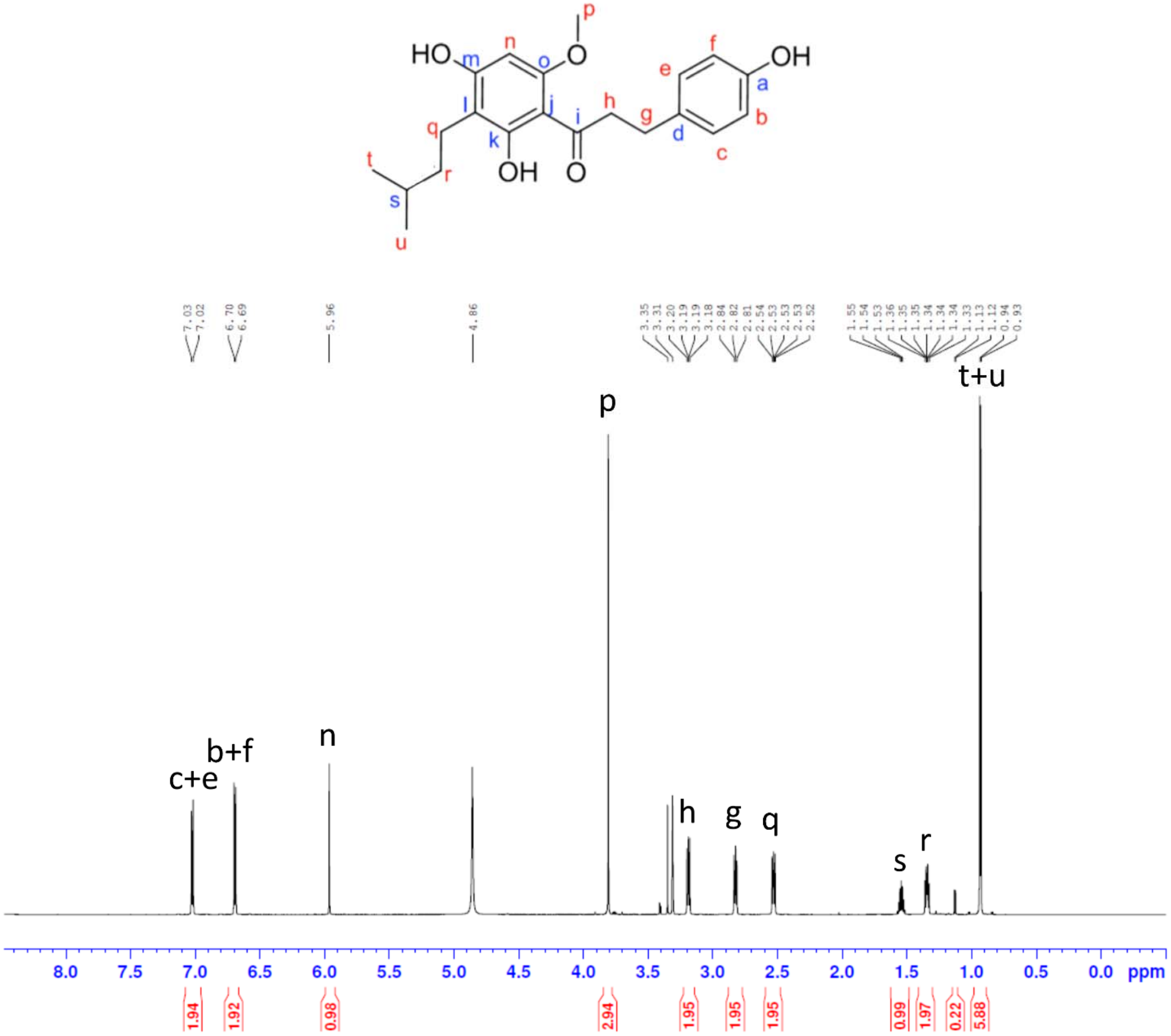

Figure S6. ^{13}C-NMR (175 MHz) spectrum of tetrahydroxanthohumol (TXN) recorded in methanol-d_4. For atom labels, see Figure S5. ^{13}C signals were assigned on the basis of HSQC and HMBC experiments.

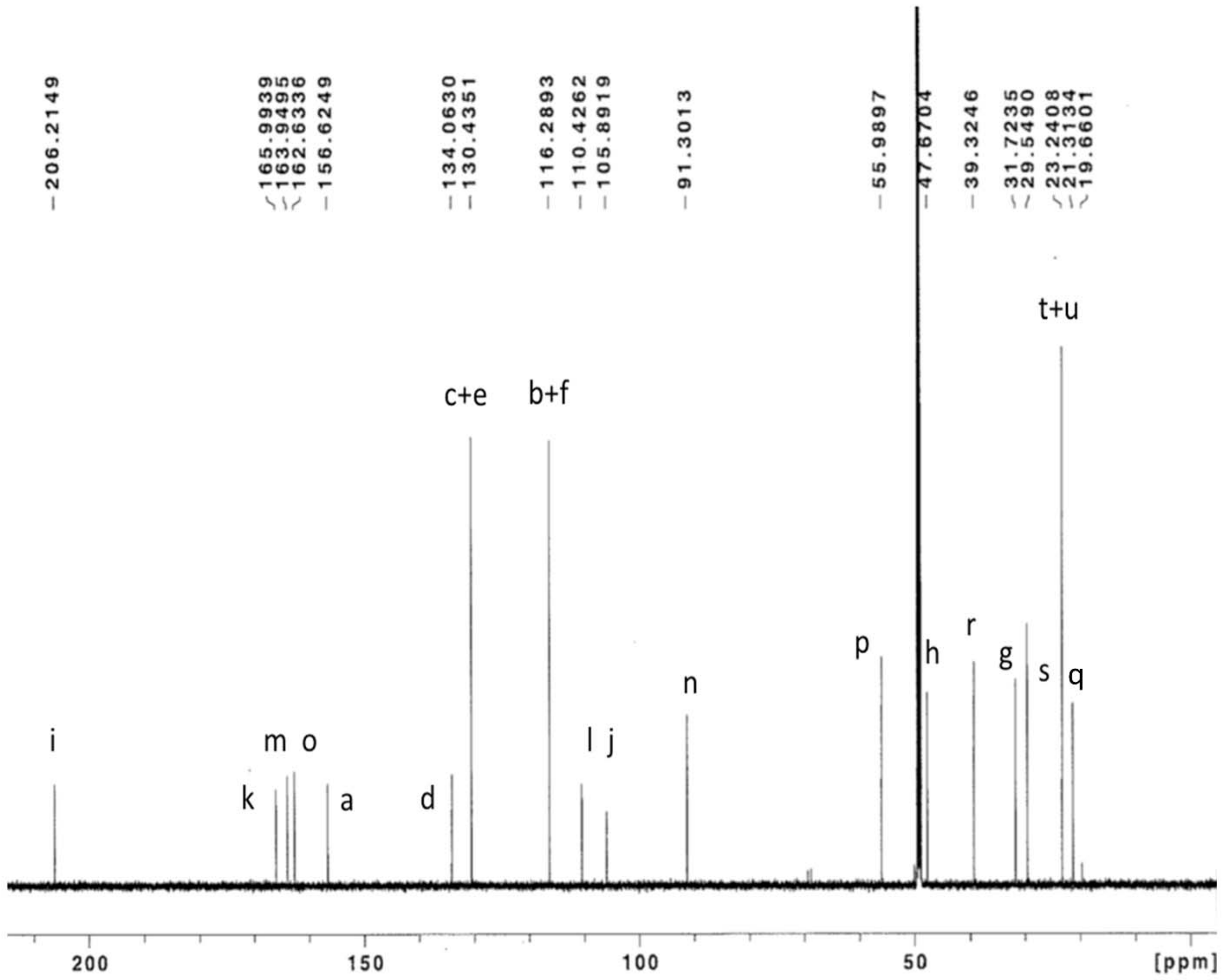

Figure S7. Electrospray MS/MS spectrum of tetrahydroxanthohumol (TXN) and its proposed fragmentation pathway.

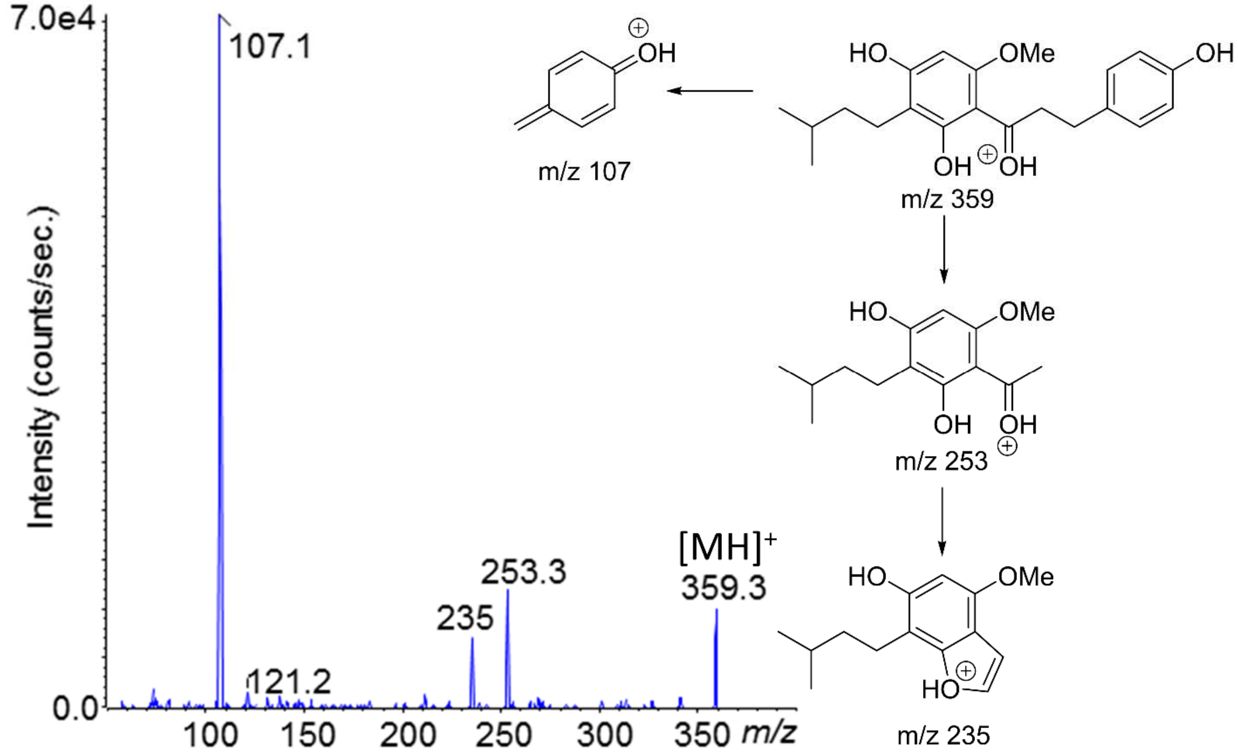

Figure S8. Full-length Western blots of liver and muscle biopsies from untreated mice (control, lanes 1-2) and treated with XN (lanes 3-4), DXN (lanes 5-6) and TXN (lanes 7-8). Tissue extracts were incubated with specific antibodies against pAMPK, AMPK and ß-actin. These original images were used to generate Figure 7. No exposure modifications were recorded.

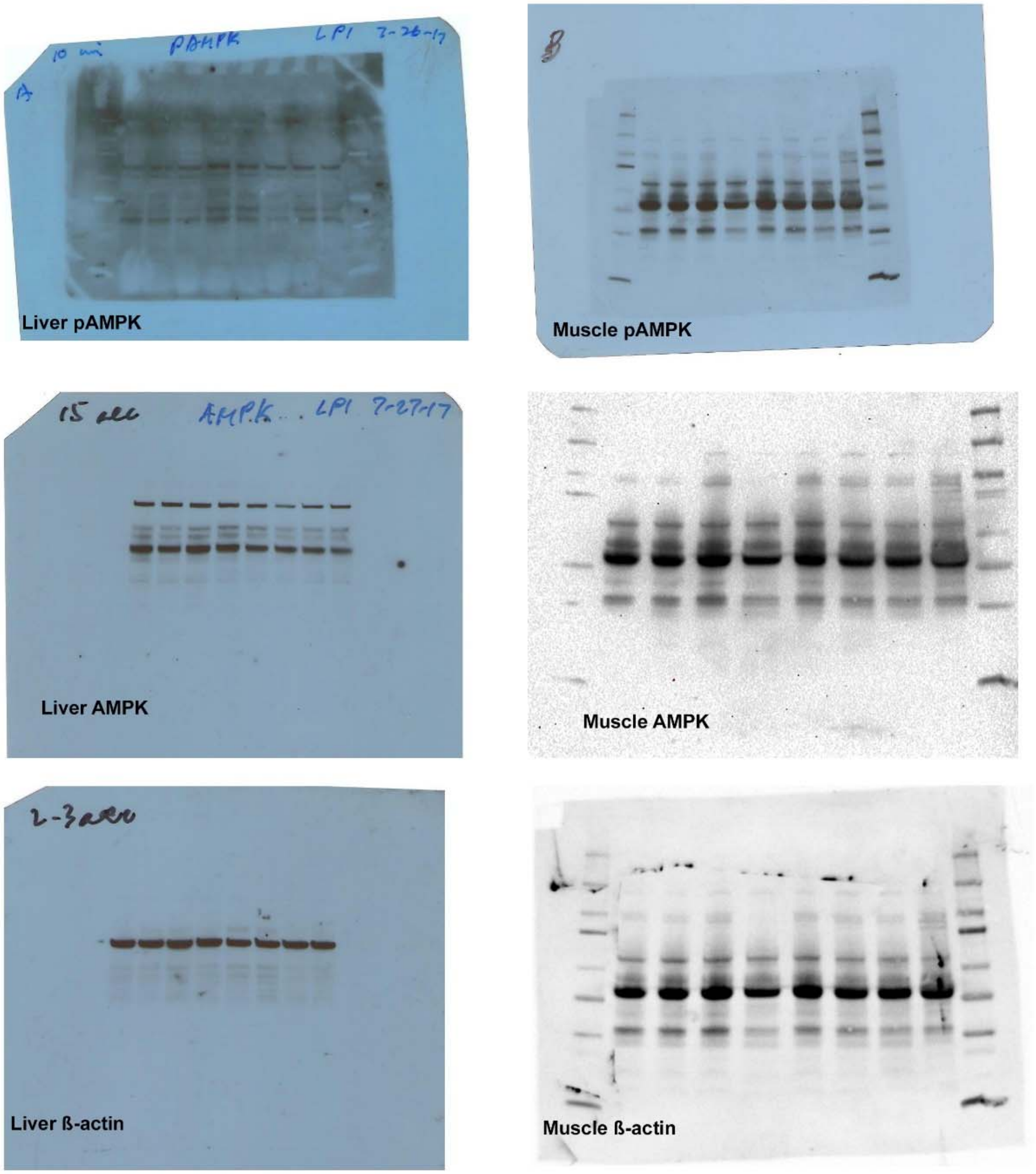

Supplement: Supplementary file 1 — Supplementary Information [file 41598_2017_18992_MOESM1_ESM.pdf]
